# Preserved ratio impaired spirometry is associated with small airway dysfunction and reduced total lung capacity

**DOI:** 10.1186/s12931-022-02216-1

**Published:** 2022-10-31

**Authors:** Ningning Zhao, Fan Wu, Jieqi Peng, Youlan Zheng, Heshen Tian, Huajing Yang, Zhishan Deng, Zihui Wang, Haiqing Li, Xiang Wen, Shan Xiao, Peiyu Huang, Cuiqiong Dai, Lifei Lu, Kunning Zhou, Shengtang Chen, Yumin Zhou, Pixin Ran

**Affiliations:** 1 State Key Laboratory of Respiratory Disease, National Clinical Research Center for Respiratory Disease, National Center for Respiratory Medicine, Guangzhou Institute of Respiratory Health, The First Affiliated Hospital of Guangzhou Medical University, 151 Yanjiang west Road, Guangzhou Laboratory, Guangzhou, China; 2 Medical Imaging Center, Wengyuan County People’s Hospital, Shaoguan, China

**Keywords:** Preserved ratio impaired spirometry, PRISm, COPD, Small airway dysfunction

## Abstract

**Background:**

Preserved ratio impaired spirometry (PRISm) refers to decreased forced expiratory volume in 1 s (FEV_1_) in the setting of preserved ratio. Little is known about the role of PRISm and its complex relation with small airway dysfunction (SAD) and lung volume. Therefore, we aimed to investigate the associations between PRISm and SAD and lung volume.

**Methods:**

We conducted a cross-sectional community-dwelling study in China. Demographic data, standard respiratory epidemiology questionnaire, spirometry, impulse oscillometry (IOS) and computed tomography (CT) data were collected. PRISm was defined as post-bronchodilator FEV_1_/FVC ≥ 0.70 and FEV_1_ < 80% predicted. Spirometry-defined SAD was defined as at least two of three of the post-bronchodilator maximal mid-expiratory flow (MMEF), forced expiratory flow 50% (FEF50), and forced expiratory flow 75% (FEF75) less than 65% of predicted. IOS-defined SAD and CT-defined gas trapping were defined by the fact that the cutoff value of peripheral airway resistance R5–R20 > 0.07 kPa/L/s and LAA_− 856_>20%, respectively. Analysis of covariance and logistic regression were used to determine associations between PRISm and SAD and lung volume. We then repeated the analysis with a lower limit of normal definition of spirometry criteria and FVC definition of PRISm. Moreover, we also performed subgroup analyses in ever smoker, never smoker, subjects without airway reversibility or self-reported diagnosed asthma, and subjects with CT-measured total lung capacity ≥70% of predicted.

**Results:**

The final analysis included 1439 subjects. PRISm had higher odds and more severity in spirometry-defined SAD (pre-bronchodilator: odds ratio [OR]: 5.99, 95% confidence interval [95%CI]: 3.87–9.27, P < 0.001; post-bronchodilator: OR: 14.05, 95%CI: 8.88–22.24, P < 0.001), IOS-defined SAD (OR: 2.89, 95%CI: 1.82–4.58, P < 0.001), and CT-air trapping (OR: 2.01, 95%CI: 1.08–3.72, P = 0.027) compared with healthy control after adjustment for confounding factors. CT-measured total lung capacity in PRISm was lower than that in healthy controls (4.15 ± 0.98 vs. 4.78 ± 1.05 L, P < 0.05), after adjustment. These results were robust in repeating analyses and subgroup analyses.

**Conclusion:**

Our finding revealed that PRISm was associated with SAD and reduced total lung capacity. Future studies to identify the underlying mechanisms and longitudinal progression of PRISm are warranted.

**Supplementary Information:**

The online version contains supplementary material available at 10.1186/s12931-022-02216-1.

## Introduction

Preserved ratio impaired spirometry (PRISm) refers to the phenomenon of a decrease in forced expiratory volume in 1 s (FEV_1_) while the FEV_1_/forced vital capacity (FVC) remains constant, and has previously been referred to as the Global Initiative for Chronic Obstructive Lung Disease-Unclassified (GOLD-U) [1], restrictive [2, 3], or nonspecific pattern [4]. Estimates of the cross-sectional prevalence of PRISm range from 4.7 to 22.3% in the population-based study [2–8]. Despite local and regional differences in PRISm prevalence, these estimates have remained relatively stable regardless of whether the fix ratios or lower limits of normal diagnostic criteria were used [9]. Decline of FEV_1_ is associated with respiratory symptoms, various comorbidities, functional limitations, and increased risks of cardiovascular mortality and all-cause mortality [3, 8, 10–12]. Data from cohort study suggested that 25.1% of PRISm subjects progressed to spirometry-defined chronic obstructive pulmonary disease (COPD) after 5 years in COPDGene study [10], and 32.6% of PRISm subjects progressed to spirometry-defined COPD after 4.5 years in the Rotterdam study [8]. As a result, PRISm was considered as one of the definitions of Pre-COPD, that is, a high-risk of population for COPD without spirometry-defined airflow obstruction [13].

Small airways are generally defined as airways less than 2 mm in diameter [14]. Previous comprehensive physiological studies have found that the loss of small airways predates the development of emphysema and COPD [15]. Measurement of small airway function might be used for identifying individuals at a high risk of developing chronic respiratory disease [14, 16]. There are many indirect methods for assessing small airway function, such as lung function, impulse oscillometry (IOS), computed tomography (CT), body plethysmography, inert gas washout, and Magnetic Resonance Imaging (MRI) [14]. Previous studies found that PRISm progressing to COPD was an airway-predominant disease in COPDGene cohort and speculated that PRISm individuals may have small airway obstruction along with small lungs [17, 18]. Therefore, the study of the relationship between PRISm and small airway dysfunction (SAD) and lung volume is particularly important to understand the functional and structural pathophysiological abnormalities for PRISm.

Limited data regarding the functional and structural pathophysiological abnormalities for PRISm are available. With this in mind, we examined baseline data from Early Chronic Obstructive Pulmonary Disease (ECOPD) cohort to evaluate the functional and structural pathophysiological abnormalities, to investigate the differences among healthy subjects and patients with PRISm, and to provide a theoretical basis to understand PRISm. We hypothesized that PRISm was associated with SAD and reduced total lung capacity.

## Methods

### Study design and study population

This study is based on cross-sectional data from the ECOPD cohort in Guangdong, China. Detailed ECOPD cohort methodological details have been described previously [19]. In short, subjects were continuously recruited into the group from July 2019 to December 2020 from four regions (Yuexiu District, Guangzhou City; Haizhu District, Guangzhou City; Wengyuan County, Shaoguan City; and Lianping County, Heyuan City). According to the timing of the completion of spirometry, we randomly invited one-fourth of participants whose FEV_1_/FVC was ≥ 0.70 after the use of bronchodilators. All participants with post-bronchodilator FEV_1_/FVC of < 0.70 were invited to participate. Participants invited and willing to take part in the study further underwent chest inspiratory and expiratory high-resolution CT and IOS. The main inclusion criteria included 40–80 years old; willing to participate in this study and provide written informed consent; completed questionnaire, spirometry, IOS and CT assessments. The main exclusion criteria included respiratory tract infection or aggravation within 4 weeks before screening; previous lobectomy; active cancer; active tuberculosis.

### Questionnaires

Questionnaire interviews were conducted by trained staff. The standard respiratory epidemiological questionnaire for this study was revised by the COPD Epidemiological Survey in China [20]. We classified the smoking status of the participants as never smoked, former smoking, and current smoking. The smoking index was defined as years of smoking times the number of cigarettes per day divided by 20 (pack-years). Family history of respiratory diseases incorporated parents, siblings related by blood, and sons and daughters of subjects with respiratory diseases, including chronic bronchitis, emphysema, asthma, COPD, cor pulmonale, bronchiectasis, lung cancer, interstitial lung disease, and obstructive sleep apnea/hypopnea syndrome. Biomass exposure refers to the use of biomass, mainly wood, crop residue, charcoal, grass, and dung for cooking or heating for more than 1 year, and occupational exposure history refers exposure to dust/harmful gases/harmful fumes for more than 1 year. The modified British Medical Research Council Questionnaire (mMRC), and COPD Assessment Test (CAT) were used to assess the quality of life [21, 22]. Acute respiratory event/acute exacerbation during preceding year was defined as cough, expectoration, purulent sputum, wheezing, dyspnea, at least two new symptoms, or aggravation of the original symptoms of the above five symptoms which persist for at least 48 h during the year prior to enrollment, at the same time excluding left and right cardiac dysfunction, pulmonary embolism, pneumothorax, pleural effusion, and arrhythmia [23].

### Spirometry and impulse oscillometry

Following the guidelines of the American Thoracic Society (ATS) and the European Respiratory Society (ERS) [24], trained technicians used a portable lung function instrument, the MasterScreen Pneumo PC spirometer (CareFusion, Yorba Linda, CA, USA) for spirometry to measure vital capacity before and after bronchodilator use. Spirometry was performed while sitting with a nose clip, with at least three measurements. At least two measurements with an error of less than 5% must be produced as a standard for correct performance. For the bronchodilation test, after the spirometry was completed the subjects were asked to inhale 400 µg of salbutamol in a 500-mL spacer, and the inhalation time of salbutamol was recorded. The predicted FEV_1_, FVC, maximal mid-expiratory flow (MMEF), forced expiratory flow 50% (FEF50), and forced expiratory flow 75% (FEF75) was obtained using the reference values for Chinese population [25]. Airflow reversibility was defined as an increase in FEV_1_ greater than or equal to 0.2 L, and the change in FEV_1_ is defined as greater than or equal to 12% of the measured value of the pre-bronchodilator lung function. PRISm was defined as post-bronchodilator FEV_1_/FVC ≥ 0.70 and FEV_1_ < 80% predicted. Healthy control was defined as post-bronchodilator FEV_1_/FVC ≥ 0.70 and FEV_1_ ≥ 80% predicted. Spirometry-defined COPD was defined as post-bronchodilator FEV_1_/FVC < 0.70. Spirometry-defined SAD was defined as at least two of three of the MMEF, FEF50, and FEF75 were less than 65% of predicted [26].

Impulse oscillometry (Masterscreen IOS; Jaeger, Höchberg, Germany) was carried out according to the guidelines of the manufacturers and ERS technical standards [27]. We used the Masterscreen IOS Impulse spirometer and the equations recommended by the manufacturer. The IOS system was routinely calibrated as recommended by the manufacturer. Resistances at 5 Hz (R5) and 20 Hz (R20) are used as indicators of total airway resistance and proximal airway resistance, respectively. Peripheral airway resistance refers to the decrease of resistance from 5 to 20 Hz (R5–R20, in kPa/L/s). IOS-defined SAD is defined by the fact that the cutoff value of R5–R20 is greater than 0.07 kPa/L/s [28, 29]. The reactance at 5 Hz (X5) reflects the elastic recoil of the surrounding airway, and the resonance frequency (F_res_) refers to the frequency at which the inertia characteristic of the airway is equal to the peripheral capacitance of the lung. The reactance area (A_x_, the area under the reactance curve) reflects the elastic properties of the periphery of the lungs and shows a correlation with resistance at lower frequencies [30].

### Computed tomography

CT scans of inspiratory and expiratory chest were performed in all subjects using a 128-slice helical CT scanner (Siemens Definition AS Plus and United-Imaging uCT 760) [19]. The researchers trained the participants to hold their breath at the end of a deep inhale (near total lung capacity [TLC]) and deep exhale (near residual volume [RV]) before the scan. Technicians and radiologists were double-blinded to the clinical characteristics and pulmonary function of the subjects. After completing the inspiratory and expiratory CT scans of the thorax, two radiologists evaluated the image quality, excluded the image data that produced respiratory artifacts, and gave professional diagnostic advice about the subjects’ chest CT. Chest CT were analyzed by quantitative imaging using Chest Imaging Platform (www.chestimagingplatform.org) with semi-automated 3D Slicer software [31]. Low-attenuation area of the lung with attenuation values below − 950 Hounsfield units (HU) on full-inspiration CT (LAA_− 950_) and inspiratory 15th percentile (Perc15) was used as indices to evaluate the degree of emphysema. Low-attenuation area of the lung with attenuation values below − 856 HU on full-expiration CT (LAA_− 856_) was used as a quantitative index to evaluate the degree of gas trapping [32]. We used LAA_− 856_>20% as the criterion for the severity of gas trapping [33]. Inspiratory CT total lung capacity (TLC_CT_), expiratory CT pulmonary residual volume (RV_CT_), ratio of the mean lung density of expiration and inspiration (MLD_E/I_), high-attenuation area of the lung with attenuation values of between − 600 Hounsfield units and − 250 Hounsfield units (HAA_− 600 to −250_) were obtained [32].

### Statistical analysis

A Kolmogorov–Smirnov test was used to explore whether the quantitative information accorded with normal distribution. The quantitative variables that was normal distribution were expressed by mean ± standard deviation (SD). The Quantitative variables that was not normally distributed were expressed by the median (interquartile range [IQR]). A one-way analysis of variance or Kruskal–Wallis test were used to evaluate differences, as appropriate. The classified data were expressed by frequency and percentage, and the comparison between groups was studied by chi-squared tests or Fisher’s exact tests. Analysis of covariance was used to compare the differences among PRISm, healthy control and COPD subjects, adjusted for multiple comparisons using Bonferroni correction method. The natural logarithm (ln) transformation was performed for variables that do not conform to normal distribution. Multivariate logistic regression analysis was used to estimate odds ratio [OR] for SAD among PRISm, healthy control and COPD subjects. All multivariable analyses were adjusted for age, sex, BMI, smoking status, and smoking index.

Based on the above analysis, we carried out sensitivity analysis and subgroup analysis to evaluate the robustness of our results. First, considering that the use of fixed threshold cutoffs may lead to overestimation of COPD in the elderly, we repeated the analysis with a lower limit of normal (LLN) thresholds definition of PRISm (post-bronchodilator FEV_1_/FVC ≥ LLN and FEV_1_ < LLN), COPD-LLN (post-bronchodilator FEV_1_/FVC < LLN), and healthy control-LLN (post-bronchodilator FEV_1_/FVC ≥ LLN and FEV_1_ ≥ LLN). Second, we also use FVC definition of PRISm (post-bronchodilator FEV_1_/FVC ≥ 0.70 and FVC < 80% predicted) for sensitivity analysis. Third, we performed subgroup analyses in ever smoker, never smoker, subjects without airway reversibility or self-reported diagnosed asthma, and subjects with TLC_CT_≥70% of predicted. SPSS 24.0 statistical software (IBM, Armonk, NY, USA) was used for all analyses. All tests were two-sided, and P values of less than 0.05 were considered statistically significant.

## Results

### Characteristics of subjects with PRISm

**Fig. 1:**
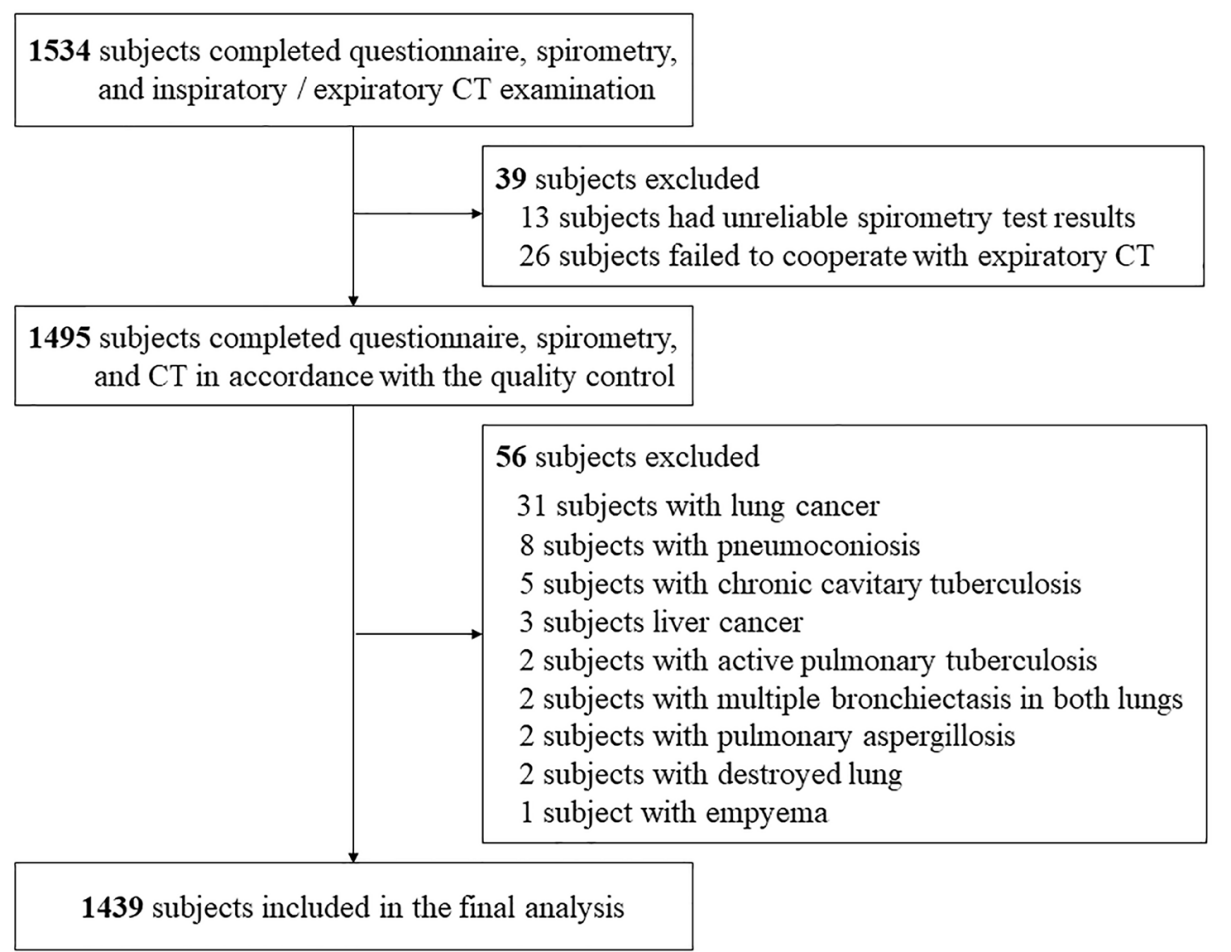
Flowchart of participants throughout the study

A flowchart outlining the selection of study participants is shown in Fig. 1. In total, 1534 subjects aged between 40 and 80 years completed respiratory epidemiology questionnaire, spirometry, and CT. After ruling out the subjects who met the exclusion criteria, a total of 1439 subjects were enrolled in the study. The clinical characteristics and spirometry of PRISm, healthy control, and spirometry-defined COPD patients are summarized in Tables 1 and 2. Of the 1439 subjects, 628 (43.6%) were healthy controls, 126 (8.8%) had PRISm, and 685 (47.6%) had COPD. Compared with spirometry-defined COPD, the PRISm was younger, with a lower proportion of males, higher BMI, more complicated with diabetes, and less positive of airflow reversibility. Compared with healthy control, the PRISm group was older, more complicated with diabetes, and had more wheezing. Compared with both groups, the FVC of PRISm subjects before and after inhaling bronchodilators was lower than that of healthy controls (pre-bronchodilator: 2.44 ± 0.58 vs. 3.27 ± 0.72 L, P < 0.05; post-bronchodilator: 2.40 ± 0.53 vs. 3.26 ± 0.70 L, P < 0.05) and COPD patients (pre-bronchodilator: 2.44 ± 0.58 vs. 3.24 ± 0.80 L, P < 0.05; post-bronchodilator: 2.40 ± 0.53 vs. 3.36 ± 0.78 L, P < 0.05). The proportion of PRISm subjects with FVC < 80% was higher in comparison with healthy controls (pre-bronchodilator: 70.6% vs. 6.1%, P < 0.05; post-bronchodilator: 72.2% vs. 3.8%, P < 0.05) and COPD (pre-bronchodilator: 70.6% vs. 22.3%, P < 0.05; post-bronchodilator: 72.2% vs. 14.9%, P < 0.05) before and after bronchodilator use.

**Table 1:** Clinical characteristics of participants by spirometric lung function class

Characteristic	Healthy control (n = 628)	PRISm (n = 126)	Spirometry-defined COPD (n = 685)
Age, years	57.8 ± 7.7	60.9 ± 8.3*^†^	64.9 ± 7.1
Male sex, n (%)	389 (61.9)	75 (59.5)^†^	631 (92.1)
Body mass index, kg/m^2^	23.6 ± 3.1	23.3 ± 3.5^†^	22.3 ± 3.3
Smoking status, n (%)			
Never smoked	318 (50.6)	68 (54.0)^†^	89 (13.0)
Former smoking	86 (13.7)	20 (15.9)^†^	218 (31.8)
Current smoking	224 (35.7)	38 (30.2)^†^	378 (55.2)
Smoking index, pack-years	19.7 ± 28.6	20.1 ± 28.3^†^	35.0 ± 30.4
Biomass exposure, n (%)	250 (39.8)	46 (36.5)	287 (41.9)
Occupational history to dusts/gases/fumes, n (%)	110 (17.5)	30 (23.8)	216 (31.5)
Family history of respiratory diseases, n (%)	63 (10.1)	16 (12.7)^†^	145 (21.3)
History of pulmonary tuberculosis, n (%)	11 (5.5)	2 (8.0)^†^	25 (28.1)
Chronic cough during childhood, n (%)	6 (18.3)	4 (3.2)	32 (4.7)
Previous medication for respiratory disease, n (%)	66(10.5)	18 (14.3)^†^	351 (51.2)
mMRC dyspnea scale score	0.2 ± 0.5	0.3 ± 0.5^†^	0.5 ± 0.7
CAT score	3.8 ± 4.2	4.5 ± 5.7^†^	5.7 ± 5.6
Acute respiratory events / exacerbations during preceding year, n (%)	21 (3.4)	9 (7.3)^†^	96 (14.0)
Self-reported diagnosed COPD, n (%)	12 (1.9)	5 (4.0)^†^	252 (36.8)
Self-reported diagnosed asthma, n (%)	3 (0.5)	1 (0.8)	21 (3.1)
**Comorbidities**, n (%)			
Hypertension	100 (15.9)	24 (19.0)	112 (16.4)
Diabetes	26 (4.1)	12 (9.5)*^†^	25 (3.6)
Coronary heart disease	24 (3.8)	4 (3.2)	24 (3.5)
Cerebral infarction	13 (2.1)	4 (3.2)	22 (3.2)
Chronic cough, n (%)	109 (17.4)	27 (21.4)^†^	296 (43.2)
Chronic phlegm, n (%)	142 (22.6)	36 (28.6)^†^	332 (48.5)
Dyspnea, n (%)	119 (19.0)	30 (23.8)^†^	280 (40.9)
Wheeze, n (%)	36 (5.7)	16 (12.7)*	130 (19.0)

Data are mean ± standard deviation or n (%)

Abbreviations: **PRISm**, preserved ratio impaired spirometry; **COPD**, chronic obstructive pulmonary disease; **mMRC**, modified British medical research council score; **CAT**, COPD assessment test

* P < 0.05 compared with healthy control after adjusting for multiple comparisons using Bonferroni correction method

† P < 0.05 compared with COPD after adjusting for multiple comparisons using Bonferroni correction method

**Table 2:** Pre-bronchodilator and post-bronchodilator spirometry parameters of participants by spirometric lung function class

Characteristic	Healthy control (n = 628)	PRISm (n = 126)	Spirometry-defined COPD (n = 685)
**Before bronchodilator use**			
FEV_1_, L	2.49 ± 0.53	1.82 ± 0.42*	1.85 ± 0.62
FEV_1_% of predicted, %	93.7 ± 11.2	70.7 ± 8.8*	68.6 ± 19.6
FVC, L	3.27 ± 0.72	2.44 ± 0.58*^†^	3.24 ± 0.80
FVC% of predicted, %	98.3 ± 12.0	75.2 ± 10.4*^†^	94.6 ± 18.7
FVC < 80%predicted, n (%)	38 (6.1)	89 (70.6)*^†^	153 (22.3)
FEV_1_/FVC, %	76.6 ± 6.3	74.9 ± 5.9^†^	56.4 ± 10.2
**After bronchodilator use**			
FEV_1_, L	2.57 ± 0.52	1.85 ± 0.38*	1.97 ± 0.61
FEV_1_% of predicted, %	96.7 ± 10.5	72.2 ± 6.8*	72.9 ± 19.0
FVC, L	3.26 ± 0.70	2.40 ± 0.53*^†^	3.36 ± 0.78
FVC% of predicted, %	97.9 ± 11.2	74.0 ± 8.4*^†^	98.0 ± 17.7
FVC < 80% predicted, n (%)	24(3.8)	91(72.2)*^†^	102 (14.9)
FEV_1_/FVC, %	79.3 ± 5.6	77.6 ± 5.8^†^	58.0 ± 9.7
**Airflow reversibility**, n (%)	33 (5.3)	9 (7.1)^†^	122 (17.8)

Data are mean ± standard deviation or n (%)

Abbreviations: **PRISm**, preserved ratio impaired spirometry; **COPD**, chronic obstructive pulmonary disease; **FEV**_**1**_, forced expiratory volume in one second; **FVC**, forced vital capacity

* P < 0.05 compared with healthy control adjusting for multiple comparisons using Bonferroni correction method

† P < 0.05 compared with COPD adjusting for multiple comparisons using Bonferroni correction method

### PRISm was associated with small airway dysfunction

**Fig. 2:**
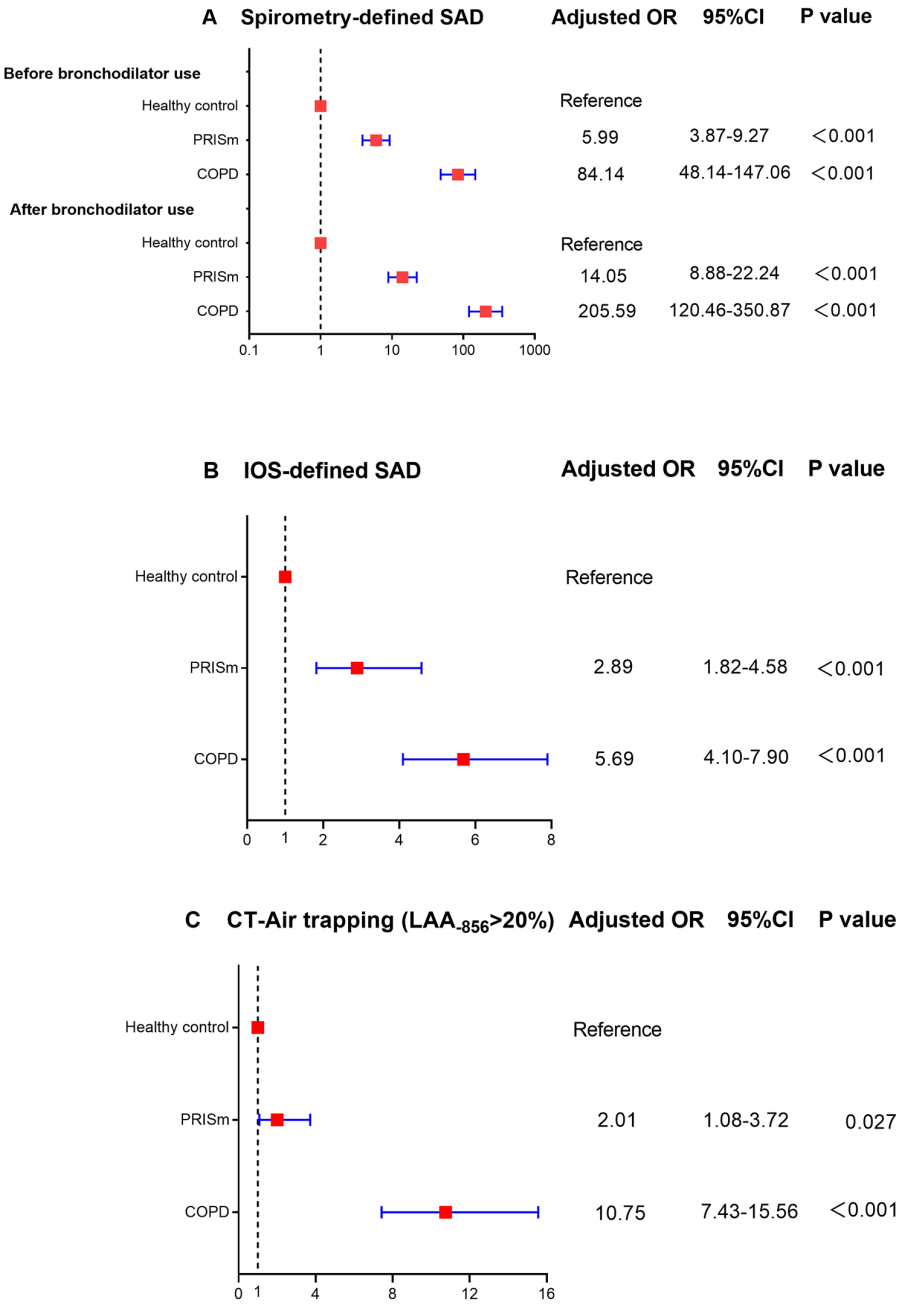
Effect of PRISm on small airway dysfunction parameters expressed as odds ratio and 95% confidence intervals by fixed ratio-defined lung function categories. Abbreviations: PRISm, preserved ratio impaired spirometry; SAD, small airway dysfunction; OR, odds ratio; CI, confidence interval; GOLD, Global Initiative for Chronic Obstructive Lung Disease; IOS, Impulse oscillometry; CT, computed tomography; LAA_-856_, low-attenuation area of the lung with attenuation values below -856 Hounsfield units. Analyses were adjusted for age, sex, body mass index, smoking status, and smoking index. P value is a result of comparison with the healthy control group.

Compared with healthy control after adjustment for age, sex, BMI, smoking status, and smoking index, the lung function parameters of SAD (MMEF, FEF50, and FEF75) in PRISm before and after bronchodilator use were lower (P < 0.05 for all comparisons). SAD-related IOS parameters (R5-R20, AX, X5, and Fres) was significantly greater in PRISm (P < 0.05 for all comparisons) compared with healthy control. Air trapping-related CT parameters (MLD_E/I_ and RV/TLC_CT_) was significantly greater in PRISm but LAA_− 856_ was no difference compared with healthy control (Table 3). Multivariable logistic regression showed that PRISm possessed more pre-bronchodilator spirometry-defined SAD (OR 5.99, 95% confidence interval [CI]: 3.87–9.27, P < 0.001), post-bronchodilator spirometry-defined SAD (OR 14.05, 95% CI: 8.88–22.24, P < 0.001), IOS-defined SAD (OR 2.89, 95% CI: 1.82–4.58, P < 0.001), and CT-air trapping (OR 2.01, 95% CI: 1.08–3.72, P = 0.027) compare with healthy control after adjustment for age, sex, BMI, smoking status, and smoking index (Fig. 2).

**Table 3:** Comparison of lung function, impulse oscillometry, and radiographic measurements of subjects by fixed ratio-defined lung function categories

Parameter	Healthy control (n = 628)	PRISm (n = 126)	Spirometry-defined COPD (n = 685)
**Lung Function**			
**Before bronchodilator use**			
MMEF, L/s	2.01 ± 0.84	1.29 ± 0.54*^†^	0.78 ± 0.40
MMEF% of predicted, %	80.6 ± 29.4	54.1 ± 19.0*^†^	31.9 ± 15.1
FEF50, L/s	2.76 ± 1.04	1.84 ± 0.74*^†^	1.07 ± 0.60
FEF50% of predicted, %	83.7 ± 27.6	57.8 ± 19.5*^†^	32.7 ± 17.1
FEF75, L/s	0.66 ± 0.39	0.42 ± 0.23*^†^	0.26 ± 0.14
FEF75% of predicted, %	70.9 ± 36.4	47.5 ± 21.4*^†^	30.2 ± 15.2
**After bronchodilator use**			
MMEF, L/s	2.32 ± 0.85	1.51 ± 0.60*^†^	0.86 ± 0.41
MMEF% of predicted, %	93.3 ± 29.4	63.7 ± 23.7*^†^	34.9 ± 15.0
FEF50, L/s	3.13 ± 1.03	2.07 ± 0.72*^†^	1.19 ± 0.62
FEF50% of predicted, %	95.1 ± 27.1	65.5 ± 20.3*^†^	36.3 ± 17.3
FEF75, L/s	0.80 ± 0.43	0.52 ± 0.32*^†^	0.28 ± 0.14
FEF75% of predicted, %	85.6 ± 41.8	59.4 ± 33.9*^†^	32.8 ± 15.1
**Impulse oscillometry**§			
R5^¶^, kPa/L/s	0.31 ± 0.09	0.36 ± 0.11*^†^	0.37 ± 0.13
R20^¶^, kPa/L/s	0.27 ± 0.07	0.29 ± 0.07*^†^	0.28 ± 0.07
R5-R20^¶^, kPa/L/s^‡^	0.04 (0.02 to 0.06)	0.06 (0.03 to 0.10)*^†^	0.07 (0.03 to 0.14)
AX, kPa/L^‡^	0.24 (0.15 to 0.40)	0.44 (0.27 to 0.73)*^†^	0.56 (0.23 to 1.51)
X5, kPa/L/s	-0.09 ± 0.04	-0.12 ± 0.04*^†^	-0.15 ± 0.10
Fres, Hz	12.39 ± 3.45	15.24 ± 4.31*^†^	17.81 ± 6.82
**Radiographic measurements**			
LAA_− 950_, %^‡^	0.31 (0.11 to 0.78)	0.25 (0.09 to 0.66)*^†^	2.00 (0.67 to 5.92)
Perc 15, HU	-905 ± 20	-895 ± 30*^†^	-920 ± 24
LAA_− 856_, %^‡^	3.55 (1.11 to 8.42)	5.08 (1.87 to 11.6)^†^	25.87 (11.66 to 45.19)
HAA_− 600 to −250_, %^‡^	3.55 (3.19 to 4.06)	3.80 (3.42 to 4.66)^*†^	3.56 (3.16 to 4.09)
MLD_E/I_	0.82 ± 0.06	0.86 ± 0.06*^†^	0.91 ± 0.06
TLC_CT_, L	4.78 ± 1.05	4.15 ± 0.98*^†^	5.45 ± 1.13
TLC_CT_ % of predicted, %	87.7 ± 22.0	76.7 ± 22.2*^†^	117.7 ± 35.4
RV_CT_, L^‡^	2.17 (1.82 to 2.59)	2.15 (1.81 to 2.52)^†^	3.29 (2.68 to 4.06)
RV_CT_ % of predicted, %^‡^	103.1 (85.2 to 123.4)	101.4 (83.0 to 120.7)^†^	160.4 (123.9 to 201.1)
RV/TLC_CT_^‡^	0.46 (0.40 to 0.52)	0.52 (0.46 to 0.63)*^†^	0.61 (0.51 to 0.74)

Data are mean ± standard deviation or median (interquartile range)

Abbreviations: **PRISm**, preserved ratio impaired spirometry; **COPD**: chronic obstructive pulmonary disease; **MMEF**, maximal mid-expiratory flow; **FEF50**, forced expiratory flow 50%; **FEF75**, forced expiratory flow 75%; **R5**, Resistances at 5 Hz; **R20**, Resistances at 20 Hz; **R5-R20**: Resistances at 5 and 20 Hz; **Ax**, Reactance area; **X5**, Reactance at 5 Hz; **Fres**, Resonant frequency in Hz; **LAA**_**− 950**_, low-attenuation area of the lung with attenuation values below − 950 Hounsfield units; **Perc 15**, 15th percentile; **HU**, Hounsfield Unit; **LAA**_**− 856**_, low-attenuation area of the lung with attenuation values below − 856 Hounsfield units; **HAA**_**− 600 to −250**_, high-attenuation area of the lung with attenuation values of between − 600 Hounsfield units and − 250 Hounsfield units. **MLD**_**E/I**_, ratio of the mean lung density of expiration to inspiration; **TLC**_**CT**_, CT-measured total lung capacity; **RV**_**CT**_, CT-measured residual volume

* P < 0.05 compared with healthy control using analysis of covariance adjusting for multiple comparisons using Bonferroni correction method

† P < 0.05 compared with COPD using analysis of covariance adjusting for multiple comparisons using Bonferroni correction method

Outcomes were adjusted for age, sex, body mass index, smoking status, and smoking index

‡ Use the natural log (ln) of the variables that were not normally distribution

§ Numbers of subjects with impulse oscillometry available: PRISm = 115, Healthy control = 589, Spirometry-defined COPD = 643.

¶ R5, R20, and R5-R20 were used as indicators of total airway resistance, proximal airway resistance, and peripheral airway resistance respectively

### PRISm was associated with reduced total lung capacity

PRISm had significantly lower TLC_CT_ (4.15 ± 0.98 vs. 4.78 ± 1.05 L, P < 0.05) and TLC_CT_ % of predicted (76.7 ± 22.2 vs. 87.7 ± 22.0%, P < 0.05) compared with healthy control after adjustment for age, sex, BMI, smoking status, and smoking index, but there was no significant difference in RV_CT_ and RV_CT_ % of predicted (Table 3). At the same time, the TLC_CT_ (4.15 ± 0.98 vs. 5.45 ± 1.13 L, P < 0.05) and TLC_CT_ % of predicted (76.7 ± 22.2 vs. 117.7 ± 35.4%, P < 0.05) indicators of PRISm are significantly lower than those of COPD.

### Sensitivity analysis and subgroup analysis

These associations remained present when we repeated the analysis with LLN definition (**Table E1-E3** and **Figure E1**) and FVC definition of PRISm (**Table E4-E6** and **Figure E2**). Moreover, findings were similar in ever smokers and in never smokers (**Table E7-E8** and **Figure E3-E4**). Considering that asthma and positive airway reversibility can affect the measurement of small airway function, we restricted our analysis to subjects without airway reversibility or self-reported diagnosed asthma and these associations remained robust (**Table E9** and **Figure E5**). In order to eliminate the effect of low TLC_CT_ on small airway function, we analyzed the subjects with TLC_CT_≥ 70% for subgroup analysis and the results were still robust (**Table E10** and **Figure E6**). In the subgroup analysis of never smokers, there was no difference in R5-R20 and LAA_− 856_ between PRISm and healthy control. However, there were differences in other indexes reflecting SAD between the two groups, which was believed to be the reason for the small sample size of the subjects who never smoked.

## Discussion

To our knowledge, this study is the first to investigate the association between PRISm and SAD, and the proportion, clinical characteristics of PRISm in a community-dwelling population in China. Individuals with PRISm were younger, with higher BMI and higher rate of diabetes than COPD patients. Individuals with PRISm were older, higher rate of diabetes, and had more wheezing than healthy controls. The results revealed that individuals with PRISm had more severe SAD and reduced TLC_CT_ compared with healthy controls.

Small airway are difficult to directly measure because they are less than 2 mm in diameter. There are many methods to measure SAD indirectly. We used spirometry, IOS, and CT to evaluate SAD. Spirometric parameters of SAD (MMEF, FEF50, FEF75) were most practical and feasible in the community-dwelling study, but the reproducibility and comparability of the spirometric parameters of SAD were limited. IOS was considered to be more sensitive and effort-independent than spirometry, it reflected small airway function by measuring airway resistance, and was easier to perform because only normal tidal breathing was required. CT could assess not only gas trapping on the expiratory but also structural abnormalities in the lungs. We demonstrated that relationships between PRISm and SAD from three perspectives. Their relationship remained present in three perspectives, sensitivity analysis, and subgroup analysis. Therefore, the results of this study were robust.

Individuals with PRISm have more severe SAD and lower emphysema than individuals with normal spirometry. These results supported the hypothesis that PRISm was an airway-predominant disease state, although the reason for this remains unclear. Previous studies have shown that occupational exposure to dust/gases/fumes could lead to small airway obstruction [34–36], and our studies have shown that occupational exposure to dust/gases/fumes history in PRISm subjects were slightly higher than those in healthy controls. Therefore, we speculate that the above results may be related to occupational exposure to dust/gases/fumes, although this needs to be clarified by further pathophysiological studies. Moreover, COPDGene cohort studies have shown that the progression of PRISm to spirometry-defined COPD was mainly airway-predominant disease, which is different from the progression of normal spirometry to spirometry-defined COPD which was mainly emphysema-predominant disease [17]. This represents exactly two lung function trajectories leading to COPD: low FEV_1_ and accelerated decline in FEV_1_ [37, 38]. PRISm could be used as one of the clinical subtypes of Pre-COPD [13]. We will follow up individuals with PRISm in the ECOPD cohort to provide more prognostic evidences. However, we should also recognize that PRISm was an unstable state with some heterogeneity. On the basis of strengthening the screening and follow-up management of COPD, the screening and management of other possible diseases and extrapulmonary diseases should also be strengthened.

Our study found that lung volume in individuals with PRISm was significantly lower than that in healthy controls and COPD patients. This result was consistent with the COPDGene cohort [10]. Our findings have added existing knowledge to support the association between PRISm and reduced total lung capacity. This may be related to early abnormal lung growth and development, leading to a failure to achieve maximum lung volume and maximum lung function in adulthood and an eventual emergence of PRISm [39, 40]. Moreover, previous studies also found that pulmonary tuberculosis in adult could cause permanent damage to lung anatomy and was associated with spirometric restriction [41]. However, the proportion of previous pulmonary tuberculosis in the PRISm group was not significantly higher than that in healthy control in our study (Table 1), probably because the sample size was too small.

CT-defined small airway function may be better assessed using parametric response maps that combined inspiratory and expiratory CT images [42]. However, due to the inaccessibility of CT image analysis software, we were unable to obtain parametric response map data. The use of expiratory LAA_− 856_ to assess CT-defined small airway function is also one of the commonly used methods [33]. We also assessed small airway function measures of pulmonary function and small airway function measures of IOS. Therefore, the absence of parametric response maps to assess CT-defined small airway function is unlikely to have influenced the conclusions of this study.

The advantage of this study is that it is the first in China to extensively describe PRISm in terms of spirometry of the general Chinese population according to clinical and radiological variables. Another strength of this study is that we used post-bronchodilator for diagnosis of PRISm. Post-bronchodilator spirometry diagnosis of PRISm might reduce the proportion of PRISm and avoid some PRISm subjects being misdiagnosed as spirometry-defined COPD [9]. Meantime, the HUNT study found that mortality was better predicted by post-bronchodilator than by pre-bronchodilator spirometry [43].

Our research has some potential limitations that should be noted. Most importantly, this is an observational and cross-sectional study and we cannot determine causal relationship between PRISm and SAD and reduced total lung capacity. Therefore, association results in our study should be interpreted carefully. Secondly, we did not perform body plethysmography, insert gas washout, MRI on PRISm subjects because ECOPD cohort was a community-dwelling study and the above methods for assessing small airway function and lung volume are difficult to carry out in the community hospital and primary care setting. Third, because one out of four of patients with normal lung function were randomly selected for this study, the possibility of non-response bias and volunteer bias could not be ruled out. Fourth, the sample size of PRISm included in this study was limited. Differences in respiratory symptoms or comorbidities between PRISm, healthy control, and COPD groups could not be detected due to the limited sample size limiting statistical power. Fifth, this study was based on a single pulmonary function diagnosis of PRISm. Previous studies have found that PRISm is an unstable state with high volatility, and the volatility of PRISm may affect the results of this study [44].

## Conclusion

In summary, our finding demonstrated that PRISm was a common pattern of pulmonary function measurement and was underestimated in the Chinese community. This study suggested that PRISm was associated with SAD and reduced total lung capacity in comparison with individuals with normal spirometry. Individuals with PRISm should be identified early through screening, and strategies aimed at improving or controlling for PRISm should be implemented in the early stages of lung disease. Further studies are needed to explore the underlying mechanism related to the occurrence of PRISm, and a longitudinal study with a large sample size is needed to evaluate the progression of PRISm.

## Electronic supplementary material

Below is the link to the electronic supplementary material.

Supplementary Material 1

## Data Availability

The data that support the findings of this study are available on request from the corresponding author.

## Abbreviations

**AX**: Reactance area.
**BMI**: body mass index.
**CAT**: COPD assessment test.
**COPD**: chronic obstructive pulmonary disease.
**CI**: confidence interval.
**CT**: computed tomography.
**FEV**_**1**_: forced expiratory volume in one second.
**Fres**: Resonant frequency.
**FVC**: forced vital capacity.
**HU**: Hounsfield Unit.
**IOS**: Impulse Oscillometry.
**LAA**_**− 856**_: Low-attenuation area of the lung with attenuation values below − 856 Hounsfield units.
**LAA**_**− 950**_: Low-attenuation area of the lung with attenuation values below − 950 Hounsfield units.
**LLN**: lower limit of normal.
**mMRC**: modified British Medical Research Council Questionnaire.
**MLD**_**E/I**_: ratio of the mean lung density of expiration to inspiration
**HAA**_**− 600 to −250**_: high-attenuation area of the lung with attenuation values of between − 600 Hounsfield units and − 250 Hounsfield units
**OR**: odds ratio.
**Perc 15**: 15th percentile.
**PRISm**: Preserved Ratio Impaired Spirometry.
**R5**: Resistance at 5 Hz.
**R20**: Resistance at 20 Hz.
**R5-R20**: Difference between resistance at 5 and 20 Hz.
**RV**_**CT**_: CT-measured residual volume
**SAD**: small airway dysfunction.
**SE**: standard error.
**TLC**_**CT**_: CT-measured total lung capacity
**X5**: Reactance at 5 Hz.
