# Supplementary material for: Preserved ratio impaired spirometry is associated with small airway dysfunction and reduced total lung capacity
Source: Respir Res. 2022 Oct 31;23:298. doi: 10.1186/s12931-022-02216-1 (PMC9620623; doi:10.1186/s12931-022-02216-1)
Supplement: Supplementary file 1 — Supplementary Material 1 [file 12931_2022_2216_MOESM1_ESM.docx]

**Online Data supplement**

**Preserved ratio impaired spirometry is associated with small airway dysfunction and reduced total lung capacity**

**Running title**: PRISm and SAD.

**Authors:** Ningning Zhao^1†^, MD; Fan Wu^1†^, MD; Jieqi Peng^1†^, MD; Youlan Zheng^1^, MD; Heshen Tian^1^, MD, PhD; Huajing Yang^1^, MD; Zhishan Deng^1^, MD; Zihui Wang^1^, MD; Haiqing Li^1^, MD; Xiang Wen^1^, MD; Shan Xiao^1^, MD; Peiyu Huang^1^, MD; Cuiqiong Dai^1^, MD; Lifei Lu^1^, MD; Kunning Zhou^1^, MD; Shengtang Chen^2^, MD; Yumin Zhou^1*^, MD, PhD; Pixin Ran^1*^, MD, PhD.

**Institutions and Affiliations:**

^1^ State Key Laboratory of Respiratory Disease, National Clinical Research Center for Respiratory Disease, National Center for Respiratory Medicine, Guangzhou Institute of Respiratory Health, The First Affiliated Hospital of Guangzhou Medical University, Guangzhou Laboratory, Guangzhou, China.

^2^ Medical Imaging Center, Wengyuan County People's Hospital, Shaoguan, China.

† **These authors contributed equally to this work.**

* **Corresponding Author**

Prof. **Pixin Ran**.

State Key Laboratory of Respiratory Disease, National Clinical Research Center for Respiratory Disease, National Center for Respiratory Medicine, Guangzhou Institute of Respiratory Health, The First Affiliated Hospital of Guangzhou Medical University, Guangzhou Laboratory, 151 Yanjiang west Road, Guangzhou, China.

1. mail: pxran@gzhmu.edu.cn. ORCID: 0000-0001-6651-634X.

Prof. **Yumin Zhou.**

State Key Laboratory of Respiratory Disease, National Clinical Research Center for Respiratory Disease, National Center for Respiratory Medicine, Guangzhou Institute of Respiratory Health, The First Affiliated Hospital of Guangzhou Medical University, Guangzhou Laboratory, 151 Yanjiang west Road, Guangzhou, China.

E-mail: zhouyumin410@126.com. ORCID: 0000-0002-0555-8391.

**Contents**

**Table E1.** Clinical characteristics of participants by LLN defined lung function categories.

**Table E2.** Pre-bronchodilator and post-bronchodilator spirometry parameters of participants by LLN defined lung function categories.

**Table E3.** Comparison of lung function, impulse oscillometry, and radiographic measurements of subjects by LLN defined lung function categories.

**Table E4.** Clinical characteristics of participants in subgroups PRISm defined using FEV1/FVC≥0.70 and FVC<80% predicted.

**Table E5.** Pre-bronchodilator and post-bronchodilator spirometry parameters of participants in subgroups PRISm defined using FEV_1_/FVC≥0.70 and FVC<80% predicted.

**Table E6.** Comparison of lung function category, impulse oscillometry, and radiographic measurements of PRISm, healthy control and COPD in subgroups PRISm defined using FEV_1_/FVC≥0.70 and FVC<80% predicted.

**Table E7.** Comparison of lung function category, radiographic measurements and impulse oscillometry of PRISm, healthy control and COPD in never smokers.

**Table E8.** Comparison of lung function category, impulse oscillometry, and radiographic measurements of PRISm, healthy control and COPD in former and current smokers.

**Table E9.** Comparison of lung function category, impulse oscillometry, and radiographic measurements of PRISm, healthy control and COPD in subgroups without airway reversibility or self-reported diagnosed asthma.

**Table E10.** Comparison of lung function category, impulse oscillometry, and radiographic measurements of PRISm, healthy control and COPD in subgroups with TLC_CT_≥70% of the predicted value.

**Figure E1**. Effect of PRISm on small airway dysfunction parameters expressed as odds ratio and 95% confidence intervals by lower limit of normal-defined lung function categories.

**Figure E2**. Effect of PRISm on small airway dysfunction parameters expressed as odds ratio and 95% confidence intervals in subgroups PRISm defined using FEV_1_/FVC≥0.70 and FVC<80% predicted.

**Figure E3**. Effect of PRISm on small airway dysfunction parameters expressed as odds ratio and 95% confidence intervals in never smokers.

**Figure E4**. Effect of PRISm on small airway dysfunction parameters expressed as odds ratio and 95% confidence intervals in former and current smokers.

**Figure E5**. Effect of PRISm on small airway dysfunction parameters expressed as odds ratio and 95% confidence intervals in subgroups without airway reversibility or self-reported diagnosed asthma.

**Figure E6**. Effect of PRISm on small airway dysfunction parameters expressed as odds ratio and 95% confidence intervals in subgroups with TLC_CT_≥70% of the predicted value.

**Table E1.** Clinical characteristics of participants by LLN defined lung function categories.

| **Characteristic** | **Healthy control-LLN** (FEV_1_/FVC≥LLN & FEV_1_ ≥LLN) | **PRISm-LLN** (FEV_1_/FVC≥LLN & FEV_1_ <LLN) | **COPD-LLN** (FEV_1_/FVC<LLN) |
| --- | --- | --- | --- |
| Number | 641 | 161 | 637 |
| Age, years | 58.4±8.0 | 61.1±8.1*^†^ | 64.6±7.2 |
| Male sex, n (%) | 403 (62.9) | 108 (67.1)^†^ | 584 (91.7) |
| Body mass index, kg/m^2^ | 23.6±3.1 | 23.4±3.4^†^ | 22.2±3.3 |
| Smoking status, n (%) |  |  |  |
| Never smoked | 314 (49.0) | 79 (49.1) ^†^ | 82 (12.9) |
| Former smoking | 99 (15.4) | 27 (16.8)^†^ | 198 (31.1) |
| Current smoking | 228 (35.6) | 55 (34.2)^†^ | 357 (56.0) |
| Smoking index, pack-years | 20.6±29.2 | 22.5±29.2^†^ | 34.2±30.1 |
| Biomass exposure, n (%) | 251 (39.2) | 65 (40.4) | 267 (41.9) |
| Occupational history to dusts/gases/fumes, n (%) | 110 (17.2) | 37 (23.0)^†^ | 209 (32.8) |
| Family history of respiratory diseases, n (%) | 63 (9.9) | 21 (13.0)^†^ | 140 (22.1) |
| History of pulmonary tuberculosis, n (%) | 12 (6.1) | 3 (7.5)^†^ | 23 (29.5) |
| Chronic cough during childhood, n (%) | 7 (1.1) | 4 (2.5) | 31 (4.9) |
| Previous medication for respiratory disease, n (%) | 74 (11.5) | 21 (13.0)^†^ | 340 (53.4) |
| mMRC dyspnea scale score | 0.2±0.5 | 0.2±0.5^†^ | 0.5±0.7 |
| CAT score | 3.9±4.2 | 3.8±5.2^†^ | 6.0±5.7 |
| Acute respiratory events / exacerbations during preceding year, n (%) | 22 (19.5) | 6 (19.4) | 97 (29.6) |
| Self-reported diagnosed COPD, n (%) | 17 (2.7) | 7 (4.3)^†^ | 245 (38.5) |
| Self-reported diagnosed asthma, n (%) | 2 (0.3) | 2 (1.2) | 21 (3.3) |
| **Comorbidities, n (%)** |  |  |  |
| Hypertension | 104 (16.2) | 34 (21.1) | 98 (15.4) |
| Diabetes | 30 (4.7) | 12 (7.5)^†^ | 21 (3.3) |
| Coronary heart disease | 24 (3.7) | 7 (4.3) | 21 (3.3) |
| Cerebral infarction | 15 (2.3) | 5 (3.1) | 19 (3.0) |
| Chronic cough, n (%) | 112 (17.5) | 32 (19.9)^†^ | 288 (45.2) |
| Chronic phlegm, n (%) | 146 (22.8) | 39 (24.2)^†^ | 325 (51.0) |
| Dyspnea, n (%) | 129 (20.1) | 34 (21.3)^†^ | 266 (41.8) |
| Wheeze, n (%) | 38 (5.9) | 16 (9.9)^†^ | 128 (20.1) |

Data are mean ± standard deviation or n (%).

Abbreviations: **LLN**, lower limit of normal; **PRISm**, preserved ratio impaired spirometry; **COPD**, chronic obstructive pulmonary disease; **mMRC**, modified British medical research council score; **CAT**, COPD assessment test.

* P<0.05 compared with healthy control after adjusting for multiple comparisons using Bonferroni correction method.

† P<0.05 compared with COPD after adjusting for multiple comparisons using Bonferroni correction method.

**Table E2.** Pre-bronchodilator and post-bronchodilator spirometry parameters of participants by LLN defined lung function categories.

| **Characteristic** | **Healthy control-LLN** (FEV_1_/FVC≥LLN& FEV_1_ ≥LLN) | **PRISm-LLN** (FEV_1_/FVC≥LLN & FEV_1_ <LLN) | **COPD-LLN** (FEV_1_/FVC<LLN) |
| --- | --- | --- | --- |
| Number | 641 | 161 | 637 |
| **Before bronchodilator use** |  |  |  |
| FEV_1_, L | 2.49±0.53 | 1.88±0.43* | 1.82±0.62 |
| FEV_1_% of predicted, % | 94.0±11.0 | 71.6±8.7*^†^ | 67.4±19.5 |
| FVC, L | 3.29±0.72 | 2.56±0.61*^†^ | 3.24±0.81 |
| FVC% of predicted, % | 99.0±12.2 | 77.3±10.6*^†^ | 94.3±18.8 |
| FVC<LLN, n (%) | 126 (19.8) | 36 (22.4) | 137 (22.1) |
| FEV_1_/FVC, % | 76.1±6.6 | 73.9±6.3*^†^ | 55.6±10.1 |
| **After bronchodilator use** |  |  |  |
| FEV_1_, L | 2.57±0.52 | 1.92±0.40* | 1.94±0.61 |
| FEV_1_% of predicted, % | 97.1±10.3 | 73.4±6.8* | 71.8±18.8 |
| FVC, L | 3.28±0.70 | 2.51±0.55*^†^ | 3.36±0.79 |
| FVC% of predicted, % | 98.8±11.3 | 76.1±8.7*^†^ | 97.9±17.8 |
| FVC<LLN, n (%) | 30 (4.7) | 114 (71.3)*^†^ | 108 (17.0) |
| FEV_1_/FVC, % | 78.8±5.9 | 76.8±5.9*^†^ | 57.1±9.6 |
| **Airflow reversibility, n (%)** | 35 (5.5) | 14 (8.7)^†^ | 115 (18.1) |

Data are mean ± standard deviation or n (%).

Abbreviations: **LLN**, lower limit of normal; **PRISm**, preserved ratio impaired spirometry; **COPD**, chronic obstructive pulmonary disease; **FEV_1_**, forced expiratory volume in one second; **FVC**, forced vital capacity.

* P<0.05 compared with healthy control after adjusting for multiple comparisons using Bonferroni correction method.

† P<0.05 compared with COPD after adjusting for multiple comparisons using Bonferroni correction method.

**Table E3.** Comparison of lung function, impulse oscillometry, and radiographic measurements of subjects by LLN defined lung function categories.

| **Characteristic** | **Healthy control-LLN** (FEV_1_/FVC≥LLN & FEV_1_ ≥LLN) | **PRISm-LLN** (FEV_1_/FVC≥LLN & FEV_1_ <LLN) | **COPD-LLN** (FEV_1_/FVC<LLN) |
| --- | --- | --- | --- |
| Number | 641 | 161 | 637 |
| **Lung Function** |  |  |  |
| **Before bronchodilator use** |  |  |  |
| MMEF, L/s | 1.98±0.85 | 1.29±0.55*^†^ | 0.76±0.40 |
| MMEF% of predicted, % | 79.7±29.6 | 52.6±19.3*^†^ | 30.8±14.8 |
| FEF50, L/s | 2.72±1.05 | 1.83±0.77*^†^ | 1.03±0.60 |
| FEF50% of predicted, % | 82.8±27.7 | 56.3±20.2*^†^ | 31.4±16.7 |
| FEF75, L/s | 0.65±0.39 | 0.42±0.22*^†^ | 0.26±0.14 |
| FEF75% of predicted, % | 70.1±36.5 | 46.2±21.1*^†^ | 29.3±14.8 |
| **After bronchodilator use** |  |  |  |
| MMEF, L/s | 2.28±0.60 | 1.51±0.60*^†^ | 0.83±0.40 |
| MMEF% of predicted, % | 92.2±29.8 | 61.1±23.9*^†^ | 33.5±14.4 |
| FEF50, L/s | 3.09±1.05 | 2.03±0.75*^†^ | 1.14±0.60 |
| FEF50% of predicted, % | 94.4±27.4 | 63.1±20.5*^†^ | 34.5±16.4 |
| FEF75, L/s | 0.78±0.43 | 0.51±0.30*^†^ | 0.28±0.14 |
| FEF75% of predicted, % | 84.3±42.0 | 56.8±32.3*^†^ | 31.8±14.9 |
| **Impulse oscillometry**§ |  |  |  |
| R5^¶^, kPa/L/s | 0.31±0.09 | 0.35±0.11*^†^ | 0.38±0.13 |
| R20^¶^, kPa/L/s | 0.27±0.07 | 0.28±0.07*^†^ | 0.28±0.07 |
| R5-R20^¶^, kPa/L/s^‡^ | 0.04 (0.02 to 0.06) | 0.06 (0.03 to 0.10)*^†^ | 0.07 (0.03 to 0.14) |
| AX, kPa/L^‡^ | 0.23 (0.15 to 0.40) | 0.43 (0.26 to 0.72)*^†^ | 0.59 (0.24 to 1.58) |
| X5, kPa/L/s | -0.09±0.04 | -0.12±0.04*^†^ | -0.15±0.10 |
| Fres, Hz | 12.43±3.60 | 15.21±4.14*^†^ | 18.04±6.89 |
| **Radiographic measurements** |  |  |  |
| LAA_-950_, %^‡^ | 0.37 (0.13 to 0.86) | 0.29 (0.11 to 0.80)*^†^ | 2.19 (0.68 to 6.37) |
| Perc 15, HU | -905±20 | -898±27*^†^ | -920±25 |
| LAA_-856_, %^‡^ | 3.94 (1.16 to 9.37) | 4.77 (1.89 to 11.82)^†^ | 27.3 (12.5 to 47.3) |
| MLD_E/I_ | 0.83±0.06 | 0.85±0.06*^†^ | 0.91±0.06 |
| TLC_CT_, L | 4.83±1.07 | 4.29±0.99*^†^ | 5.46±1.13 |
| TLC_CT_ % of predicted, % | 89.3±24.2 | 78.8±21.6*^†^ | 118.5±35.3 |
| RV_CT_, L^‡^ | 2.19 (1.84 to 2.66) | 2.20 (1.87 to 2.60)^†^ | 3.32 (2.72 to 4.15) |
| RV_CT_ % of predicted, %^‡^ | 105.0 (85.7 to 126.1) | 102.0 (86.8 to 121.7)^†^ | 162.2 (126.4 to 203.2) |
| RV/TLC_CT_^‡^ | 0.46 (0.40 to 0.53) | 0.51 (0.44 to 0.60)*^†^ | 0.62 (0.51 to 0.75) |

Data are mean ± standard deviation or median (interquartile range).

Abbreviations: **LLN**, lower limit of normal; **PRISm**, preserved ratio impaired spirometry; **COPD:** chronic obstructive pulmonary disease; **MMEF**, maximal mid-expiratory flow; **FEF50**, forced expiratory flow 50%; **FEF75**, forced expiratory flow 75%; **R5**, Resistances at 5 Hz; **R20**, Resistances at 20 Hz; **R5-R20**: Resistances at 5 and 20 Hz; **Ax**, Reactance area; **X5**, Reactance at 5 Hz; **Fres**, Resonant frequency in Hz; **LAA_-950_**, low-attenuation area of the lung with attenuation values below -950 Hounsfield units; **Perc 15**, 15th percentile; **HU**, Hounsfield Unit; **LAA_-856_**, low-attenuation area of the lung with attenuation values below -856 Hounsfield units; **MLD_E/I_,** ratio of the mean lung density of expiration to inspiration; **TLC_CT_**, CT-measured total lung capacity; **RV_CT_**, CT-measured residual volume.

* P<0.05 compared with healthy control using analysis of covariance adjusting for multiple comparisons using Bonferroni correction method.

† P<0.05 compared with COPD using analysis of covariance adjusting for multiple comparisons using Bonferroni correction method.

Outcomes were adjusted for age, sex, body mass index, smoking status, and smoking index.

‡ Use the natural log (ln) of the variables that were not normally distribution.

§ Numbers of subjects with impulse oscillometry available: PRISm-LLN=149, Healthy control-LLN=601, COPD-LLN=597.

¶ R5, R20, and R5-R20 were used as indicators of total airway resistance, proximal airway resistance, and peripheral airway resistance respectively.

**Table E4.** Clinical characteristics of participants in subgroups PRISm defined using FEV1/FVC≥0.70 and FVC<80% predicted.

| **Parameter** | **Healthy control**  (n=639) | **PRISm**  (n=115) | **Spirometry-defined COPD** (n=685) |
| --- | --- | --- | --- |
| Age, years | 57.9±7.7 | 60.3±8.5*^†^ | 64.8±7.1 |
| Male sex, n (%) | 402 (62.9) | 62 (53.9)^†^ | 631 (92.1) |
| Body mass index, kg/m^2^ | 23.6±3.1 | 23.4±3.5^†^ | 22.3±3.3 |
| Smoking status, n (%) |  |  |  |
| Never smoked | 320 (50.1) | 66 (57.4)^†^ | 89 (13.0) |
| Former smoking | 89 (13.9) | 17 (14.8)^†^ | 218 (31.8) |
| Current smoking | 230 (36.0) | 32 (27.8)^†^ | 378 (55.2) |
| Smoking index, pack-years | 19.8±28.3 | 19.2±29.4^†^ | 35.0±30.4 |
| Biomass exposure, n (%) | 256 (40.1) | 40 (34.8) | 287 (41.9) |
| Occupational history to dusts/gases/fumes, n (%) | 115 (18.0) | 25 (21.7)^†^ | 216 (31.5) |
| Family history of respiratory diseases, n (%) | 65 (10.3) | 14 (12.2)^†^ | 145 (21.3) |
| History of pulmonary tuberculosis, n (%) | 13 (6.6) | 0 (0)^†^ | 25 (28.1) |
| Chronic cough during childhood, n (%) | 7 (1.1) | 3 (2.6) | 32 (4.7) |
| Previous medication for respiratory disease, n (%) | 69 (10.8) | 15 (13.0)^†^ | 351 (51.2) |
| mMRC dyspnea scale score | 0.2±0.5 | 0.3±0.6^†^ | 0.5±0.7 |
| CAT score | 3.9±4.3 | 4.5±5.5^†^ | 5.7±5.6 |
| Acute respiratory events / exacerbations during preceding year, n (%) | 25 (4.0) | 5 (4.4)^†^ | 96 (14.0) |
| Self-reported diagnosed COPD, n (%) | 15 (2.3) | 2 (1.7)^†^ | 252 (36.8) |
| Self-reported diagnosed asthma, n (%) | 3 (0.5) | 1 (0.9) | 21 (3.1) |
| **Comorbidities, n (%)** |  |  |  |
| Hypertension | 104 (16.3) | 20 (17.4) | 112 (16.4) |
| Diabetes | 26 (4.1) | 12 (10.4)*^†^ | 25 (3.6) |
| Coronary heart disease | 23 (3.6) | 5 (4.3) | 24 (3.5) |
| Cerebral infarction | 14 (2.2) | 3 (2.6) | 22 (3.2) |
| Chronic cough, n (%) | 114 (17.8) | 22 (19.1)^†^ | 296 (43.2) |
| Chronic phlegm, n (%) | 151 (23.6) | 27 (23.5)^†^ | 332 (48.5) |
| Dyspnea, n (%) | 119 (18.7) | 30 (26.1)^†^ | 280 (40.9) |
| Wheeze, n (%) | 37 (5.8) | 15 (13.0)* | 130 (19.0) |

Data are mean ± standard deviation or n (%).

Abbreviations: **PRISm**, preserved ratio impaired spirometry; **COPD**, chronic obstructive pulmonary disease; **mMRC**, modified British medical research council score; **CAT**, COPD assessment test.

* P<0.05 compared with healthy control after adjusting for multiple comparisons using Bonferroni correction method.

† P<0.05 compared with COPD after adjusting for multiple comparisons using Bonferroni correction method.

**Table E5.** Pre-bronchodilator and post-bronchodilator spirometry parameters of participants in subgroups PRISm defined using FEV_1_/FVC≥0.70 and FVC<80% predicted.

| **Parameter** | **Healthy control**  (n=639) | **PRISm**  (n=115) | **Spirometry-defined COPD** (n=685) |
| --- | --- | --- | --- |
| **Before bronchodilator use** |  |  |  |
| FEV_1_, L | 2.48±0.53 | 1.83±0.47* | 1.85±0.62 |
| FEV_1_% of predicted, % | 93.1±11.8 | 71.8±10.7* | 68.6±19.5 |
| FVC, L | 3.27±0.70 | 2.35±0.57*^†^ | 3.24±0.80 |
| FVC% of predicted, % | 98.2±11.9 | 73.4±9.6*^†^ | 94.6±18.7 |
| FEV_1_/FVC, % | 76.0±6.2 | 77.9±6.8^†^ | 56.4±10.2 |
| **After bronchodilator use** |  |  |  |
| FEV_1_, L | 2.56±0.53 | 1.86±0.43* | 1.97±0.61 |
| FEV_1_% of predicted, % | 96.1±11.2 | 73.4±8.9* | 73.0±19.0 |
| FVC, L | 3.26±0.68 | 2.30±0.50*^†^ | 3.36±0.78 |
| FVC% of predicted, % | 97.9±11.0 | 71.9±7.0*^†^ | 98.1±17.7 |
| FEV_1_/FVC, % | 78.7±5.4 | 81.2±6.5*^†^ | 58.0±9.7 |
| **Airflow reversibility, n (%)** | 33 (5.2) | 9 (7.8)^†^ | 122 (17.8) |

Data are mean ± standard deviation or n (%).

Abbreviations: **PRISm**, preserved ratio impaired spirometry; **COPD**, chronic obstructive pulmonary disease; **FEV_1_**, forced expiratory volume in one second; **FVC**, forced vital capacity.

* P<0.05 compared with healthy control after adjusting for multiple comparisons using Bonferroni correction method.

† P<0.05 compared with COPD after adjusting for multiple comparisons using Bonferroni correction method.

**Table E6.** Comparison of lung function category, impulse oscillometry, and radiographic measurements of PRISm, healthy control and COPD in subgroups PRISm defined using FEV_1_/FVC≥0.70 and FVC<80% predicted.

| **Parameter** | **Healthy control**  (n=639) | **PRISm**  (n=115) | **Spirometry-defined COPD** (n=685) |
| --- | --- | --- | --- |
| **Lung Function** |  |  |  |
| **Before bronchodilator use** |  |  |  |
| MMEF, L/s | 1.96±0.83 | 1.53±0.85*^†^ | 0.78±0.40 |
| MMEF% of predicted, % | 78.4±29.3 | 64.0±28.4*^†^ | 31.9±15.0 |
| FEF50, L/s | 2.69±1.03 | 2.14±1.04*^†^ | 1.07±0.60 |
| FEF50% of predicted, % | 81.6±27.7 | 67.2±27.0*^†^ | 32.7±17.1 |
| FEF75, L/s | 0.64±0.37 | 0.51±0.42*^†^ | 0.26±0.14 |
| FEF75% of predicted, % | 68.8±35.4 | 57.6±34.8*^†^ | 30.2±15.2 |
| **After bronchodilator use** |  |  |  |
| MMEF, L/s | 2.26±0.84 | 1.77±0.93*^†^ | 0.86±0.41 |
| MMEF% of predicted, % | 90.7±29.2 | 75.3±34.3*^†^ | 34.9±15.0 |
| FEF50, L/s | 3.06±1.03 | 2.40±1.06*^†^ | 1.19±0.62 |
| FEF50% of predicted, % | 92.7±27.5 | 76.3±29.0*^†^ | 36.3±17.3 |
| FEF75, L/s | 0.77±0.42 | 0.64±0.46*^†^ | 0.28±0.14 |
| FEF75% of predicted, % | 82.6±40.8 | 73.9±46.0*^†^ | 32.8±15.1 |
| **Impulse oscillometry**§ |  |  |  |
| R5^¶^, kPa/L/s | 0.32±0.09 | 0.36±0.10*^†^ | 0.37±0.13 |
| R20^¶^, kPa/L/s | 0.27±0.07 | 0.29±0.08^†^ | 0.28±0.07 |
| R5-R20^¶^, kPa/L/s^‡^ | 0.04 (0.02 to 0.06) | 0.06 (0.04 to 0.09)*^†^ | 0.07 (0.03 to 0.14) |
| AX, kPa/L^‡^ | 0.24 (0.15 to 0.41) | 0.42 (0.28 to 0.68)*^†^ | 0.56 (0.23 to 1.51) |
| X5, kPa/L/s | -0.09±0.04 | -0.11±0.04^†^ | -0.15±0.10 |
| Fres, Hz | 12.47±3.57 | 15.10±3.99*^†^ | 17.81±6.82 |
| **Radiographic measurements** |  |  |  |
| LAA_-950_, %^‡^ | 0.35 (0.13 to 0.80) | 0.14 (0.07 to 0.43)*^†^ | 2.00 (0.67 to 5.92) |
| Perc 15, HU | -905±20 | -891±28*^†^ | -920±24 |
| LAA_-856_, %^‡^ | 3.70 (1.21 to 8.83) | 3.65 (0.81 to 9.60)^†^ | 25.87 (11.66 to 45.19) |
| MLD_E/I_ | 0.83±0.06 | 0.85±0.07*^†^ | 0.91±0.06 |
| TLC_CT_, L | 4.80±1.03 | 3.98±1.00*^†^ | 5.45±1.13 |
| TLC_CT_ % of predicted, % | 88.2±21.9 | 72.7±20.8*^†^ | 117.7±35.4 |
| RV_CT_, L^‡^ | 2.18 (1.84 to 2.60) | 2.04 (1.72 to 2.39)^†^ | 3.29 (2.68 to 4.06) |
| RV_CT_ % of predicted, %^‡^ | 103.8 (86.0 to 124.6) | 94.8 (80.9 to 117.8)^†^ | 160.4 (123.9 to 201.1) |
| RV/TLC_CT_^‡^ | 0.46 (0.40 to 0.53) | 0.51 (0.45 to 0.63)*^†^ | 0.61 (0.51 to 0.74) |

Data are mean ± standard deviation or median (interquartile range).

Abbreviations: **PRISm**, preserved ratio impaired spirometry; **COPD:** chronic obstructive pulmonary disease; **MMEF**, maximal mid-expiratory flow; **FEF50**, forced expiratory flow 50%; **FEF75**, forced expiratory flow 75%; **R5**, Resistances at 5 Hz; **R20**, Resistances at 20 Hz; **R5-R20**: Resistances at 5 and 20 Hz; **Ax**, Reactance area; **X5**, Reactance at 5 Hz; **Fres**, Resonant frequency in Hz; **LAA_-950_**, low-attenuation area of the lung with attenuation values below -950 Hounsfield units; **Perc 15**, 15th percentile; **HU**, Hounsfield Unit; **LAA_-856_**, low-attenuation area of the lung with attenuation values below -856 Hounsfield units; **MLD_E/I_,** ratio of the mean lung density of expiration to inspiration; **TLC_CT_**, CT-measured total lung capacity; **RV_CT_**, CT-measured residual volume.

* P<0.05 compared with healthy control using analysis of covariance adjusting for multiple comparisons using Bonferroni correction method.

† P<0.05 compared with COPD using analysis of covariance adjusting for multiple comparisons using Bonferroni correction method.

Outcomes were adjusted for age, sex, body mass index, smoking status, and smoking index.

‡ Use the natural log (ln) of the variables that were not normally distribution.

§ Numbers of subjects with impulse oscillometry available: PRISm=105, Healthy control=599, COPD=643.

¶ R5, R20, and R5-R20 were used as indicators of total airway resistance, proximal airway resistance, and peripheral airway resistance respectively.

**Table E7.** Comparison of lung function category, radiographic measurements and impulse oscillometry of PRISm, healthy control and COPD in never smokers.

| **Parameter** | **Healthy control**  (n=318) | **PRISm**  (n=68) | **Spirometry-defined COPD** (n=89) |
| --- | --- | --- | --- |
| **Lung Function** |  |  |  |
| **Before bronchodilator use** |  |  |  |
| MMEF, L/s | 1.94±0.83 | 1.23±0.55*^†^ | 0.76±0.37 |
| MMEF% of predicted, % | 85.2±31.1 | 55.9±19.4*^†^ | 35.0±16.0 |
| FEF50, L/s | 2.63±1.01 | 1.74±0.75*^†^ | 1.05±0.57 |
| FEF50% of predicted, % | 86.7±28.7 | 58.9±20.4*^†^ | 36.2±18.6 |
| FEF75, L/s | 0.65±0.36 | 0.39±0.22*^†^ | 0.24±0.13 |
| FEF75% of predicted, % | 75.5±37.6 | 48.2±20.0*^†^ | 31.2±16.5 |
| **After bronchodilator use** |  |  |  |
| MMEF, L/s | 2.28±0.85 | 1.46±0.65*^†^ | 0.85±0.39 |
| MMEF% of predicted, % | 100.1±30.1 | 67.1±26.1*^†^ | 39.0±15.5 |
| FEF50, L/s | 3.04±1.03 | 1.96±0.77*^†^ | 1.19±0.58 |
| FEF50% of predicted, % | 100.4±27.9 | 67.0±22.4*^†^ | 40.7±17.1 |
| FEF75, L/s | 0.81±0.43 | 0.51±0.34*^†^ | 0.27±0.13 |
| FEF75% of predicted, % | 94.2±43.4 | 63.6±38.4*^†^ | 34.8±16.2 |
| **Impulse oscillometry**§ |  |  |  |
| R5^¶^, kPa/L/s | 0.35±0.09 | 0.39±0.10*^†^ | 0.44±0.16 |
| R20^¶^, kPa/L/s | 0.30±0.07 | 0.32±0.07 | 0.32±0.09 |
| R5-R20^¶^, kPa/L/s^‡^ | 0.04 (0.02 to 0.07) | 0.06 (0.03 to 0.11)^†^ | 0.09 (0.04 to 0.18) |
| AX, kPa/L^‡^ | 0.27 (0.16 to 0.46) | 0.46 (0.31 to 0.93)*^†^ | 0.83 (0.29 to 1.98) |
| X5, kPa/L/s | -0.09±0.04 | -0.12±0.05*^†^ | -0.17±0.13 |
| Fres, Hz | 12.66±3.51 | 15.74±4.59*^†^ | 18.52±6.81 |
| **Radiographic measurements** |  |  |  |
| LAA_-950_, %^‡^ | 0.16 (0.07 to 0.41) | 0.16 (0.05 to 0.35)^†^ | 0.67 (0.22 to 2.46) |
| Perc 15, HU | -900±22 | -888±34*^†^ | -910±25 |
| LAA_-856_, %^‡^ | 2.36 (0.74 to 6.54) | 3.92 (0.78 tob8.37)^†^ | 14.47 (6.70 to 32.35) |
| MLD_E/I_ | 0.82±0.06 | 0.86±0.07*^†^ | 0.90±0.06 |
| TLC_CT_, L | 4.23±0.93 | 3.71±0.92*^†^ | 4.52±1.18 |
| TLC_CT_ % of predicted, % | 77.8±19.4 | 68.6±21.1*^†^ | 94.1±30.8 |
| RV_CT_, L^‡^ | 4.07 (3.57 to 4.76) | 3.67 (3.26 to 4.03)^†^ | 4.35 (3.52 to 5.20) |
| RV_CT_ % of predicted, %^‡^ | 90.8 (80.1 to 108.0) | 93.6 (76.9 to 108.8)^†^ | 121.9 (104.5 to 156.9) |
| RV/TLC_CT_^‡^ | 0.44 (0.39 to 0.54) | 0.52 (0.45 to 0.63)*^†^ | 0.58 (0.47 to 0.72) |

Data are mean ± standard deviation or median (interquartile range).

Abbreviations: **PRISm**, preserved ratio impaired spirometry; **COPD:** chronic obstructive pulmonary disease; **MMEF**, maximal mid-expiratory flow; **FEF50**, forced expiratory flow 50%; **FEF75**, forced expiratory flow 75%; **R5**, Resistances at 5 Hz; **R20**, Resistances at 20 Hz; **R5-R20**: Resistances at 5 and 20 Hz; **Ax**, Reactance area; **X5**, Reactance at 5 Hz; **Fres**, Resonant frequency in Hz; **LAA_-950_**, low-attenuation area of the lung with attenuation values below -950 Hounsfield units; **Perc 15**, 15th percentile; **HU**, Hounsfield Unit; **LAA_-856_**, low-attenuation area of the lung with attenuation values below -856 Hounsfield units; **MLD_E/I_,** ratio of the mean lung density of expiration to inspiration; **TLC_CT_**, CT-measured total lung capacity; **RV_CT_**, CT-measured residual volume.

* P<0.05 compared with healthy control using analysis of covariance adjusting for multiple comparisons using Bonferroni correction method.

† P<0.05 compared with COPD using analysis of covariance adjusting for multiple comparisons using Bonferroni correction method.

Outcomes were adjusted for age, sex and body mass index.

‡ Use the natural log (ln) of the variables that were not normally distribution.

§ Numbers of subjects with impulse oscillometry available: PRISm=61, Healthy control=292, COPD=82.

¶ R5, R20, and R5-R20 were used as indicators of total airway resistance, proximal airway resistance, and peripheral airway resistance respectively.

**Table E8.** Comparison of lung function category, impulse oscillometry, and radiographic measurements of PRISm, healthy control and COPD in former and current smokers.

| **Parameter** | **Healthy control**  (n=310) | **PRISm**  (n=58) | **Spirometry-defined COPD** (n=596) |
| --- | --- | --- | --- |
| **Lung Function** |  |  |  |
| **Before bronchodilator use** |  |  |  |
| MMEF, L/s | 2.08±0.84 | 1.36±0.53*^†^ | 0.79±0.41 |
| MMEF% of predicted, % | 76.0±26.7 | 51.9±18.6*^†^ | 31.5±14.9 |
| FEF50, L/s | 2.90±1.05 | 1.96±0.72*^†^ | 1.07±0.61 |
| FEF50% of predicted, % | 80.6±26.0 | 56.4±18.5*^†^ | 32.1±16.8 |
| FEF75, L/s | 0.68±0.41 | 0.44±0.22*^†^ | 0.26±0.14 |
| FEF75% of predicted, % | 66.3±34.6 | 46.7±23.1*^†^ | 30.0±15.0 |
| **After bronchodilator use** |  |  |  |
| MMEF, L/s | 2.36±0.85 | 1.56±0.55*^†^ | 0.86±0.41 |
| MMEF% of predicted, % | 86.2±26.8 | 59.8±20.1*^†^ | 34.3±14.9 |
| FEF50, L/s | 3.22±1.03 | 2.20±0.64*^†^ | 1.19±0.62 |
| FEF50% of predicted, % | 89.8±25.3 | 63.7±17.7 | 35.6±17.2 |
| FEF75, L/s | 0.79±0.43 | 0.52±0.28*^†^ | 0.29±0.14 |
| FEF75% of predicted, % | 76.8±38.2 | 54.5±27.2*^†^ | 32.5±14.9 |
| **Impulse oscillometry**§ |  |  |  |
| R5^¶^, kPa/L/s | 0.31±0.09 | 0.36±0.11*^†^ | 0.37±0.13 |
| R20^¶^, kPa/L/s | 0.27±0.07 | 0.29±0.07 | 0.28±0.07 |
| R5-R20^¶^, kPa/L/s^‡^ | 0.04 (0.02 to 0.06) | 0.06 (0.03 to 0.10)* | 0.07 (0.03 to 0.14) |
| AX, kPa/L^‡^ | 0.24 (0.15 to 0.40) | 0.44 (0.27 to 0.73)* | 0.56 (0.23 to 1.51) |
| X5, kPa/L/s | -0.09±0.04 | -0.12±0.04^†^ | -0.15±0.10 |
| Fres, Hz | 12.39±3.45 | 15.24±4.31*^†^ | 17.81±6.82 |
| **Radiographic measurements** |  |  |  |
| LAA_-950_, %^‡^ | 0.55 (0.27 to 1.09) | 0.42 (0.14 to 1.03)^†^ | 2.30 (0.79 to 6.53) |
| Perc 15, HU | -910±17 | -904±21^†^ | -922±24 |
| LAA_-856_, %^‡^ | 4.76 (1.72 to 10.33) | 5.68 (2.47 to 14.06)^†^ | 27.73 (12.72 to 47.14) |
| MLD_E/I_ | 0.83±0.06 | 0.85±0.05*^†^ | 0.91±0.06 |
| TLC_CT_, L | 5.36±0.83 | 4.65±0.80*^†^ | 5.59±1.05 |
| TLC_CT_ % of predicted, % | 97.9±19.8 | 86.1±19.8*^†^ | 121.3±34.7 |
| RV_CT_, L^‡^ | 2.51 (2.12 to 2.83) | 2.36 (2.04 to 2.84)^†^ | 3.40 (2.78 to 4.18) |
| RV_CT_ % of predicted, %^‡^ | 114.7 (99.7 to 134.4) | 113.5 (94.2 to 130.8)^†^ | 166.7 (130.6 to 207.1) |
| RV/TLC_CT_^‡^ | 0.46 (0.40 to 0.52) | 0.51 (0.46 to 0.62)*^†^ | 0.62 (0.51 to 0.74) |

Data are mean ± standard deviation or median (interquartile range).

Abbreviations: **PRISm**, preserved ratio impaired spirometry; **COPD:** chronic obstructive pulmonary disease; **MMEF**, maximal mid-expiratory flow; **FEF50**, forced expiratory flow 50%; **FEF75**, forced expiratory flow 75%; **R5**, Resistances at 5 Hz; **R20**, Resistances at 20 Hz; **R5-R20**: Resistances at 5 and 20 Hz; **Ax**, Reactance area; **X5**, Reactance at 5 Hz; **Fres**, Resonant frequency in Hz; **LAA_-950_**, low-attenuation area of the lung with attenuation values below -950 Hounsfield units; **Perc 15**, 15th percentile; **HU**, Hounsfield Unit; **LAA_-856_**, low-attenuation area of the lung with attenuation values below -856 Hounsfield units; **MLD_E/I_,** ratio of the mean lung density of expiration to inspiration; **TLC_CT_**, CT-measured total lung capacity; **RV_CT_**, CT-measured residual volume.

* P<0.05 compared with healthy control using analysis of covariance adjusting for multiple comparisons using Bonferroni correction method.

† P<0.05 compared with COPD using analysis of covariance adjusting for multiple comparisons using Bonferroni correction method.

Outcomes were adjusted for age, sex and body mass index.

‡ Use the natural log (ln) of the variables that were not normally distribution.

§ Numbers of subjects with impulse oscillometry available: PRISm=54, Healthy control=297, COPD=561.

¶ R5, R20, and R5-R20 were used as indicators of total airway resistance, proximal airway resistance, and peripheral airway resistance respectively.

**Table E9.** Comparison of lung function category, impulse oscillometry, and radiographic measurements of PRISm, healthy control and COPD in subgroups without airway reversibility or self-reported diagnosed asthma.

| **Parameter** | **Healthy control**  (n=593) | **PRISm**  (n=116) | **Spirometry-defined COPD** (n=552) |
| --- | --- | --- | --- |
| **Lung Function** |  |  |  |
| **Before bronchodilator use** |  |  |  |
| MMEF, L/s | 2.05±0.84 | 1.31±0.55*^†^ | 0.82±0.42 |
| MMEF% of predicted, % | 82.1±29.2 | 54.5±19.1*^†^ | 33.7±15.4 |
| FEF50, L/s | 2.82±1.03 | 1.87±0.75*^†^ | 1.14±0.62 |
| FEF50% of predicted, % | 85.3±27.3 | 58.4±19.7*^†^ | 34.8±17.5 |
| FEF75, L/s | 0.68±0.39 | 0.42±0.23*^†^ | 0.27±0.14 |
| FEF75% of predicted, % | 72.1±36.5 | 47.8±21.4*^†^ | 31.6±15.9 |
| **After bronchodilator use** |  |  |  |
| MMEF, L/s | 2.34±0.86 | 1.50±0.62*^†^ | 0.87±0.42 |
| MMEF% of predicted, % | 93.9±29.5 | 63.1±24.2*^†^ | 35.5±15.3 |
| FEF50, L/s | 3.17±1.04 | 2.06±0.73*^†^ | 1.22±0.64 |
| FEF50% of predicted, % | 96.0±27.3 | 64.8±20.2*^†^ | 37.2±17.7 |
| FEF75, L/s | 0.80±0.43 | 0.52±0.33*^†^ | 0.28±0.14 |
| FEF75% of predicted, % | 85.7±41.4 | 59.0±34.8*^†^ | 33.2±15.5 |
| **Impulse oscillometry**§ |  |  |  |
| R5^¶^, kPa/L/s | 0.31±0.09 | 0.36±0.11*^†^ | 0.36±0.13 |
| R20^¶^, kPa/L/s | 0.27±0.07 | 0.29±0.07* | 0.27±0.07 |
| R5-R20^¶^, kPa/L/s^‡^ | 0.04 (0.02 to 0.06) | 0.06 (0.03 to 0.10)*^†^ | 0.06 (0.02 to 0.12) |
| AX, kPa/L^‡^ | 0.24 (0.15 to 0.39) | 0.43 (0.27 to 0.73)* | 0.47 (0.21 to 1.20) |
| X5, kPa/L/s | -0.09±0.04 | -0.12±0.04*^†^ | -0.14±0.10 |
| Fres, Hz | 12.34±3.45 | 15.23±4.38*^†^ | 17.17±6.80 |
| **Radiographic measurements** |  |  |  |
| LAA_-950_, %^‡^ | 0.31 (0.12 to 0.78) | 0.27 (0.09 to 0.68)*^†^ | 1.95 (0.67 to 5.92) |
| Perc 15, HU | -905±20 | -895±30*^†^ | -920±25 |
| LAA_-856_, %^‡^ | 3.39 (1.10 to 7.92) | 4.70 (1.77 to 12.31)^†^ | 24.55 (10.88 to 44.52) |
| MLD_E/I_ | 0.82±0.06 | 0.85±0.06*^†^ | 0.91±0.06 |
| TLC_CT_, L | 4.79±1.05 | 4.17±0.97*^†^ | 5.43±1.13 |
| TLC_CT_ % of predicted, % | 87.7±22.1 | 76.8±22.2*^†^ | 117.1±34.8 |
| RV_CT_, L^‡^ | 2.15 (1.80 to 2.59) | 2.18 (1.81 to 2.53)^†^ | 3.20 (2.67 to 3.97) |
| RV_CT_ % of predicted, %^‡^ | 102.7 (85.0 to 123.3) | 102.0 (82.3 to 121.6)^†^ | 156.6 (123.0 to 197.7) |
| RV/TLC_CT_^‡^ | 0.45 (0.40 to 0.52) | 0.52 (0.45 to 0.63)*^†^ | 0.61 (0.50 to 0.74) |

Data are mean ± standard deviation or median (interquartile range).

Abbreviations: **PRISm**, preserved ratio impaired spirometry; **COPD:** chronic obstructive pulmonary disease; **MMEF**, maximal mid-expiratory flow; **FEF50**, forced expiratory flow 50%; **FEF75**, forced expiratory flow 75%; **R5**, Resistances at 5 Hz; **R20**, Resistances at 20 Hz; **R5-R20**: Resistances at 5 and 20 Hz; **Ax**, Reactance area; **X5**, Reactance at 5 Hz; **Fres**, Resonant frequency in Hz; **LAA_-950_**, low-attenuation area of the lung with attenuation values below -950 Hounsfield units; **Perc 15**, 15th percentile; **HU**, Hounsfield Unit; **LAA_-856_**, low-attenuation area of the lung with attenuation values below -856 Hounsfield units; **MLD_E/I_,** ratio of the mean lung density of expiration to inspiration; **TLC_CT_**, CT-measured total lung capacity; **RV_CT_**, CT-measured residual volume.

* P<0.05 compared with healthy control using analysis of covariance adjusting for multiple comparisons using Bonferroni correction method.

† P<0.05 compared with COPD using analysis of covariance adjusting for multiple comparisons using Bonferroni correction method.

Outcomes were adjusted for age, sex, body mass index, smoking status, and smoking index.

‡ Use the natural log (ln) of the variables that were not normally distribution.

§ Numbers of subjects with impulse oscillometry available: PRISm=105, Healthy control=556, COPD=519.

¶ R5, R20, and R5-R20 were used as indicators of total airway resistance, proximal airway resistance, and peripheral airway resistance respectively.

**Table E10.** Comparison of lung function category, impulse oscillometry, and radiographic measurements of PRISm, healthy control and COPD in subgroups with TLC_CT_≥70% of the predicted value.

| **Parameter** | **Healthy control**  (n=485) | **PRISm**  (n=74) | **Spirometry-defined COPD** (n=647) |
| --- | --- | --- | --- |
| **Lung Function** |  |  |  |
| **Before bronchodilator use** |  |  |  |
| MMEF, L/s | 2.07±0.88 | 1.35±0.55*^†^ | 0.79±0.41 |
| MMEF% of predicted, % | 79.3±29.0 | 51.8±18.6*^†^ | 31.9±14.9 |
| FEF50, L/s | 2.85±1.08 | 1.92±0.68*^†^ | 1.08±0.61 |
| FEF50% of predicted, % | 82.8±27.6 | 55.5±16.7*^†^ | 32.7±17.0 |
| FEF75, L/s | 0.68±0.41 | 0.45±0.24*^†^ | 0.26±0.14 |
| FEF75% of predicted, % | 69.3±35.7 | 46.9±22.6*^†^ | 30.1±14.7 |
| **After bronchodilator use** |  |  |  |
| MMEF, L/s | 2.38±0.90 | 1.51±0.53*^†^ | 0.86±0.42 |
| MMEF% of predicted, % | 91.0±29.3 | 58.3±16.9*^†^ | 35.0±15.1 |
| FEF50, L/s | 3.22±1.08 | 2.12±0.65*^†^ | 1.20±0.63 |
| FEF50% of predicted, % | 93.9±27.2 | 61.5±15.4*^†^ | 36.3±17.4 |
| FEF75, L/s | 0.81±0.45 | 0.49±0.24*^†^ | 0.29±0.14 |
| FEF75% of predicted, % | 82.4±40.5 | 51.0±20.4*^†^ | 32.7±14.8 |
| **Impulse oscillometry**§ |  |  |  |
| R5^¶^, kPa/L/s | 0.29±0.08 | 0.34±0.10*^†^ | 0.37±0.13 |
| R20^¶^, kPa/L/s | 0.26±0.06 | 0.27±0.06* | 0.27±0.07 |
| R5-R20^¶^, kPa/L/s^‡^ | 0.03 (0.01 to 0.06) | 0.06 (0.04 to 0.10)* | 0.07 (0.03 to 0.14) |
| AX, kPa/L^‡^ | 0.21 (0.13 to 0.36) | 0.44 (0.27 to 0.76)* | 0.54 (0.23 to 1.47) |
| X5, kPa/L/s | -0.09±0.03 | -0.12±0.04* | -0.14±0.10 |
| Fres, Hz | 12.06±3.41 | 15.48±4.39*^†^ | 17.74±6.85 |
| **Radiographic measurements** |  |  |  |
| LAA_-950_, %^‡^ | 0.42 (0.19 to 0.90) | 0.42 (0.19 to 0.99)^†^ | 2.22 (0.72 to 6.10) |
| Perc 15, HU | -910±16 | -907±16*^†^ | -922±23 |
| LAA_-856_, %^‡^ | 3.83 (1.43 to 9.14) | 5.78 (2.32 to 12.58)^†^ | 26.13 ( 12.17 to 45.89) |
| HAA_-600 to -250_, %^‡^ | 3.43 (3.14 to 3.80) | 3.63 (3.33 to 4.20) | 3.53 (3.13 to 4.01) |
| MLD_E/I_ | 0.82±0.06 | 0.85±0.05*^†^ | 0.91±0.06 |
| TLC_CT_, L | 5.17±0.83 | 4.75±0.70*^†^ | 5.58±1.02 |
| TLC_CT_ % of predicted, % | 95.9±17.8 | 90.0±17.9*^†^ | 121.1±33.4 |
| RV_CT_, L^‡^ | 2.30 (1.94 to 2.72) | 2.33 (2.08 to 2.81)^†^ | 3.34 (2.73 to 4.11) |
| RV_CT_ % of predicted, %^‡^ | 109.6 (91.8 to 128.9) | 112.8 (100.4 to 128.5)^†^ | 163.9 (127.1 to 202.7) |
| RV/TLC_CT_^‡^ | 0.44 (0.40 to 0.51) | 0.50 (0.44 to 0.55)*^†^ | 0.61 (0.50 to 0.73) |

Data are mean ± standard deviation or median (interquartile range).

Abbreviations: **PRISm**, preserved ratio impaired spirometry; **COPD:** chronic obstructive pulmonary disease; **MMEF**, maximal mid-expiratory flow; **FEF50**, forced expiratory flow 50%; **FEF75**, forced expiratory flow 75%; **R5**, Resistances at 5 Hz; **R20**, Resistances at 20 Hz; **R5-R20**: Resistances at 5 and 20 Hz; **Ax**, Reactance area; **X5**, Reactance at 5 Hz; **Fres**, Resonant frequency in Hz; **LAA_-950_**, low-attenuation area of the lung with attenuation values below -950 Hounsfield units; **Perc 15**, 15th percentile; **HU**, Hounsfield Unit; **LAA_-856_**, low-attenuation area of the lung with attenuation values below -856 Hounsfield units; **HAA_-600 to -250_**, high-attenuation area of the lung with attenuation values of between -600 Hounsfield units and -250 Hounsfield units. **MLD_E/I_,** ratio of the mean lung density of expiration to inspiration; **TLC_CT_**, CT-measured total lung capacity; **RV_CT_**, CT-measured residual volume.

* P<0.05 compared with healthy control using analysis of covariance adjusting for multiple comparisons using Bonferroni correction method.

† P<0.05 compared with COPD using analysis of covariance adjusting for multiple comparisons using Bonferroni correction method.

Outcomes were adjusted for age, sex, body mass index, smoking status, and smoking index.

‡ Use the natural log (ln) of the variables that were not normally distribution.

§ Numbers of subjects with impulse oscillometry available: PRISm=69, Healthy control=455, Spirometry-defined COPD=611.

¶ R5, R20, and R5-R20 were used as indicators of total airway resistance, proximal airway resistance, and peripheral airway resistance respectively.

**Figure E1**. Effect of PRISm on small airway dysfunction parameters expressed as odds ratio and 95% confidence intervals by lower limit of normal-defined lung function categories.


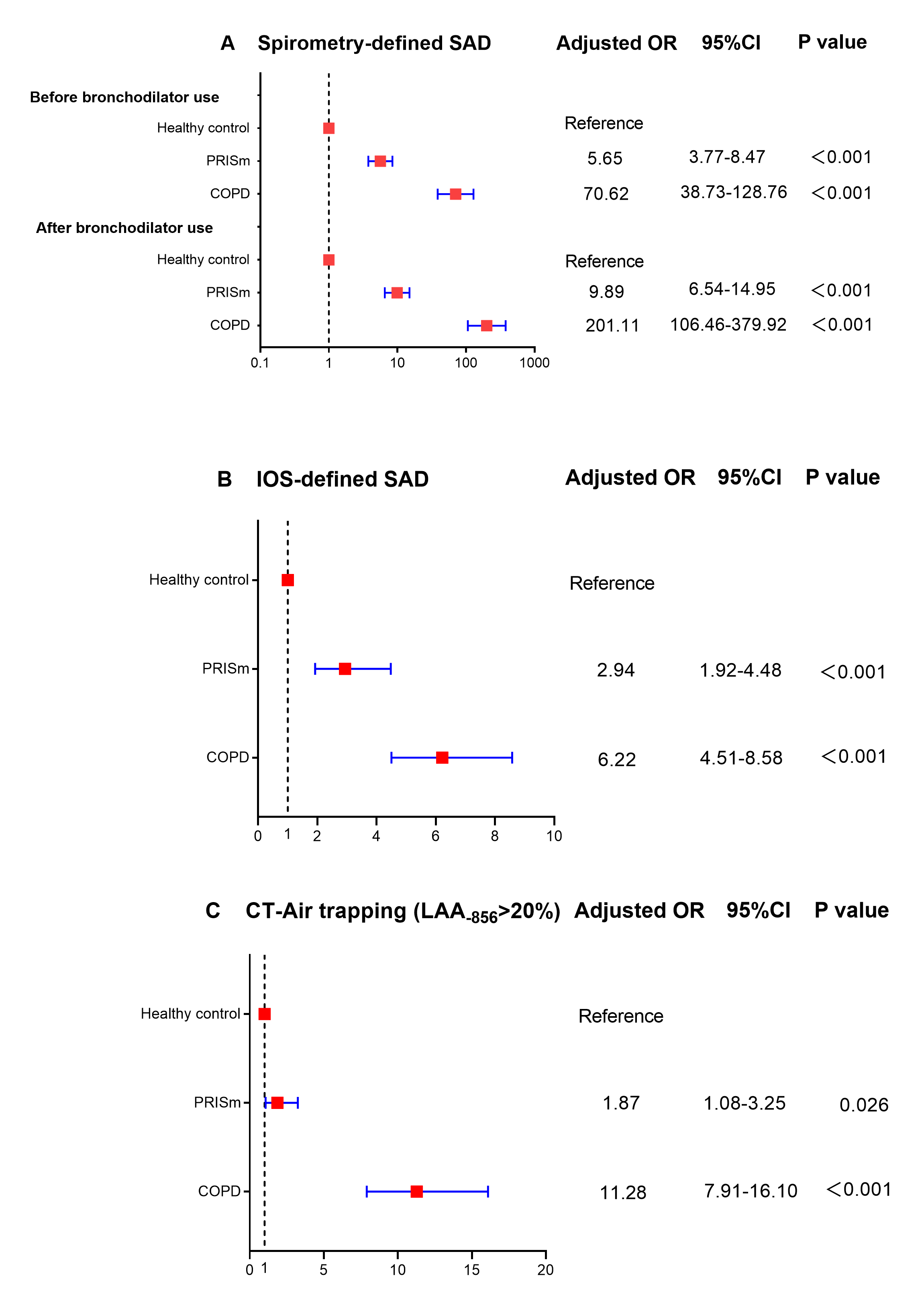


Abbreviations: PRISm, preserved ratio impaired spirometry; SAD, small airway dysfunction; OR, odds ratio; CI, confidence interval; GOLD, Global Initiative for Chronic Obstructive Lung Disease; LAA_-856_, low-attenuation area of the lung with attenuation values below -856 Hounsfield units.

Analyses were adjusted for age, sex, body mass index, smoking status, and smoking index.

P value is a result of comparison with the healthy control group.

**Figure E2**. Effect of PRISm on small airway dysfunction parameters expressed as odds ratio and 95% confidence intervals in subgroups PRISm defined using FEV_1_/FVC≥0.70 and FVC<80% predicted.


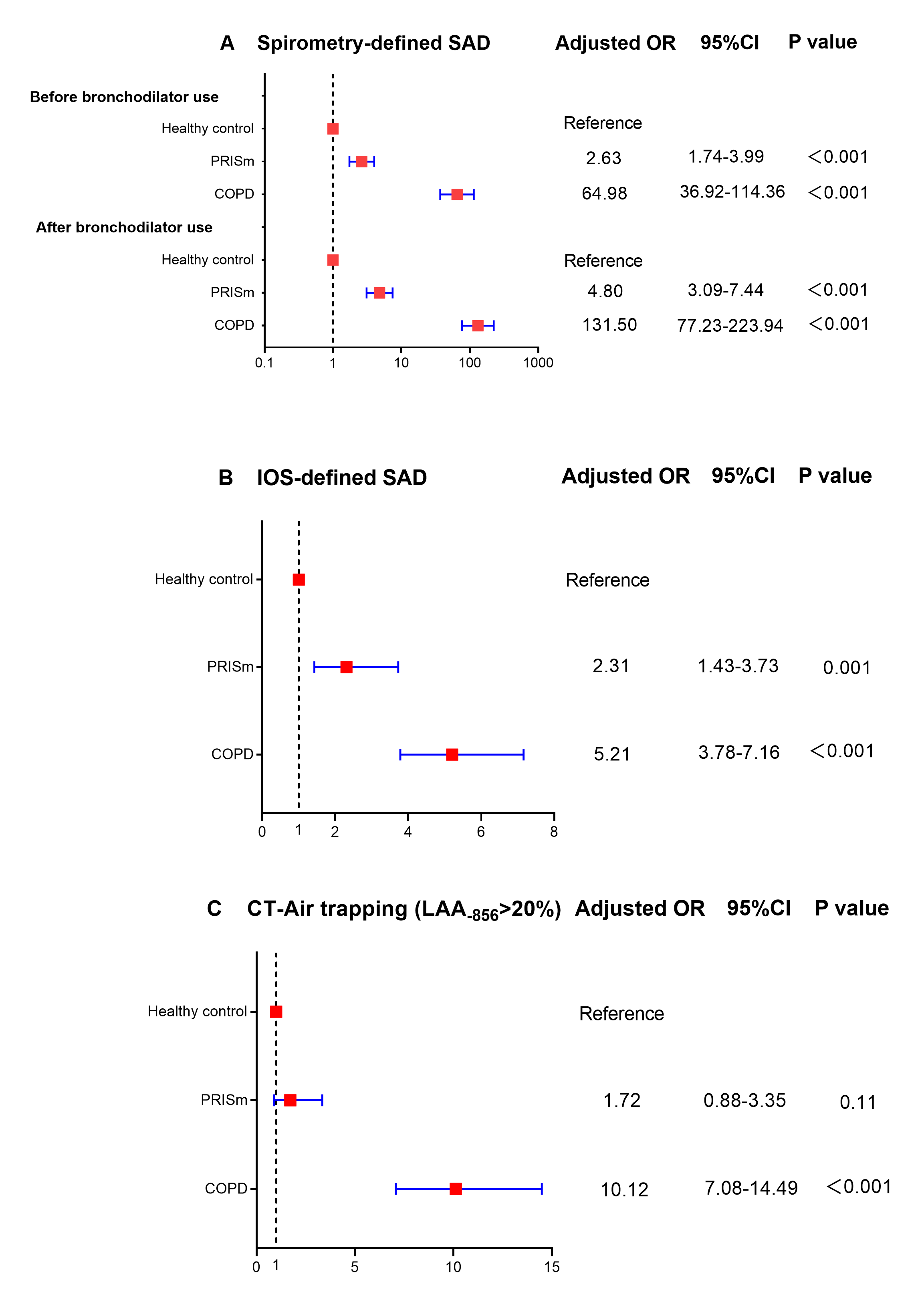


Abbreviations: PRISm, preserved ratio impaired spirometry; SAD, small airway dysfunction; OR, odds ratio; CI, confidence interval; GOLD, Global Initiative for Chronic Obstructive Lung Disease; LAA_-856_, low-attenuation area of the lung with attenuation values below -856 Hounsfield units.

Analyses were adjusted for age, sex, body mass index, smoking status, and smoking index.

P value is a result of comparison with the healthy control group.

**Figure E3**. Effect of PRISm on small airway dysfunction parameters expressed as odds ratio and 95% confidence intervals in never smokers.


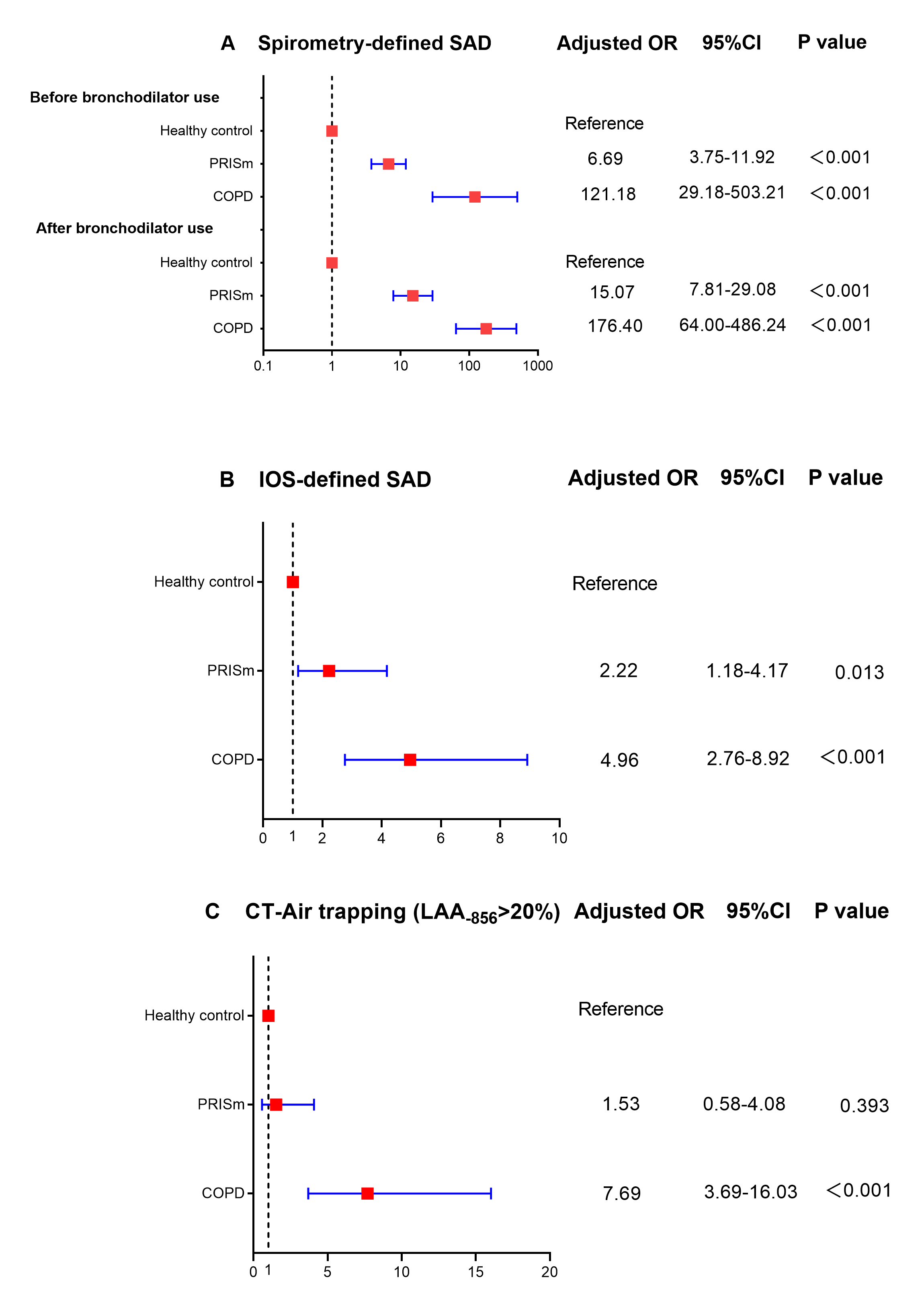


Abbreviations: PRISm, preserved ratio impaired spirometry; SAD, small airway dysfunction; OR, odds ratio; CI, confidence interval; GOLD, Global Initiative for Chronic Obstructive Lung Disease; LAA_-856_, low-attenuation area of the lung with attenuation values below -856 Hounsfield units.

Analyses were adjusted for age, sex and body mass index.

P value is a result of comparison with the healthy control group.

**Figure E4**. Effect of PRISm on small airway dysfunction parameters expressed as odds ratio and 95% confidence intervals in former and current smokers.


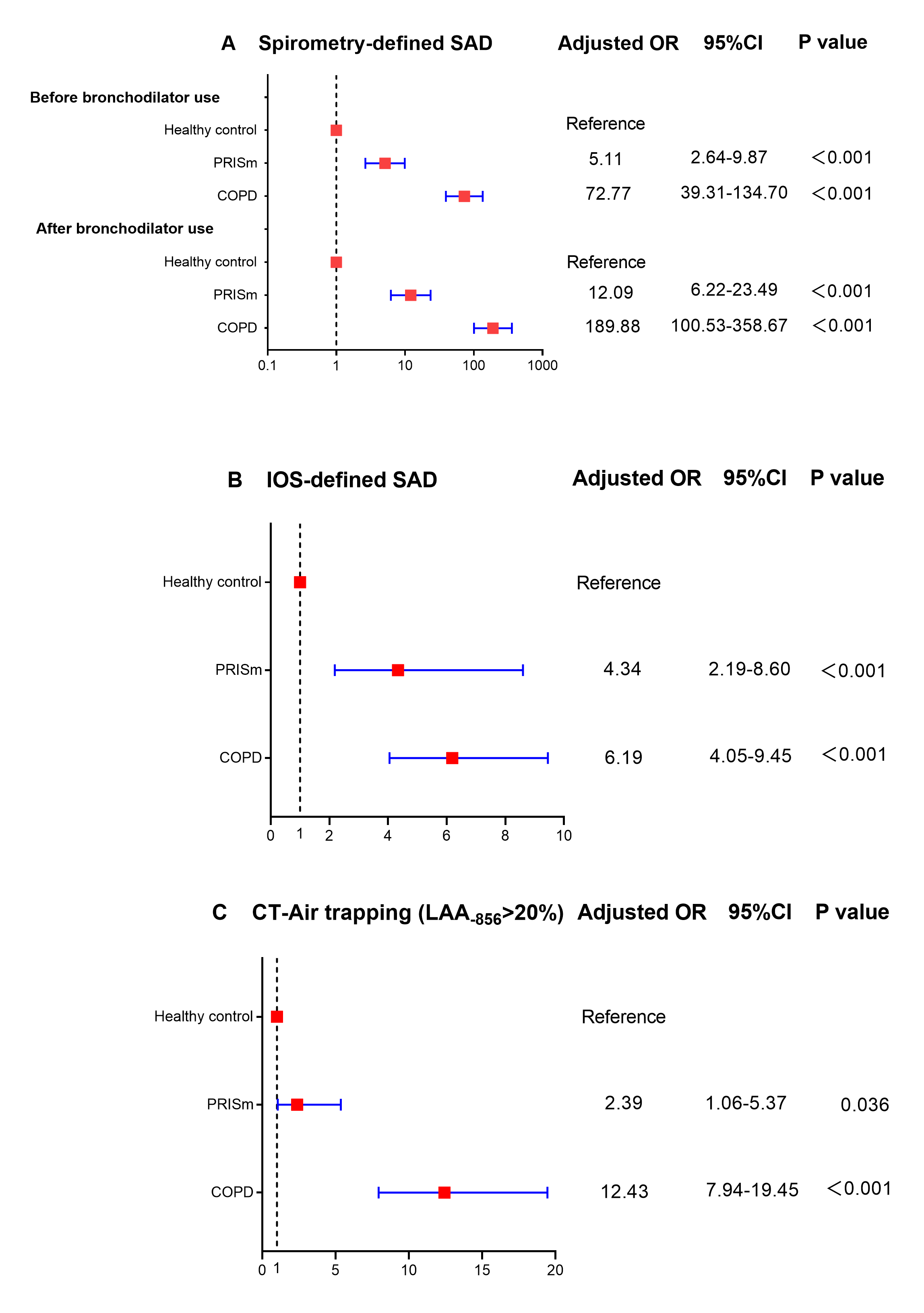


Abbreviations: PRISm, preserved ratio impaired spirometry; SAD, small airway dysfunction; OR, odds ratio; CI, confidence interval; GOLD, Global Initiative for Chronic Obstructive Lung Disease; LAA_-856_, low-attenuation area of the lung with attenuation values below -856 Hounsfield units.

Analyses were adjusted for age, sex and body mass index.

P value is a result of comparison with the healthy control group.

**Figure E5**. Effect of PRISm on small airway dysfunction parameters expressed as odds ratio and 95% confidence intervals in subgroups without airway reversibility or self-reported diagnosed asthma.


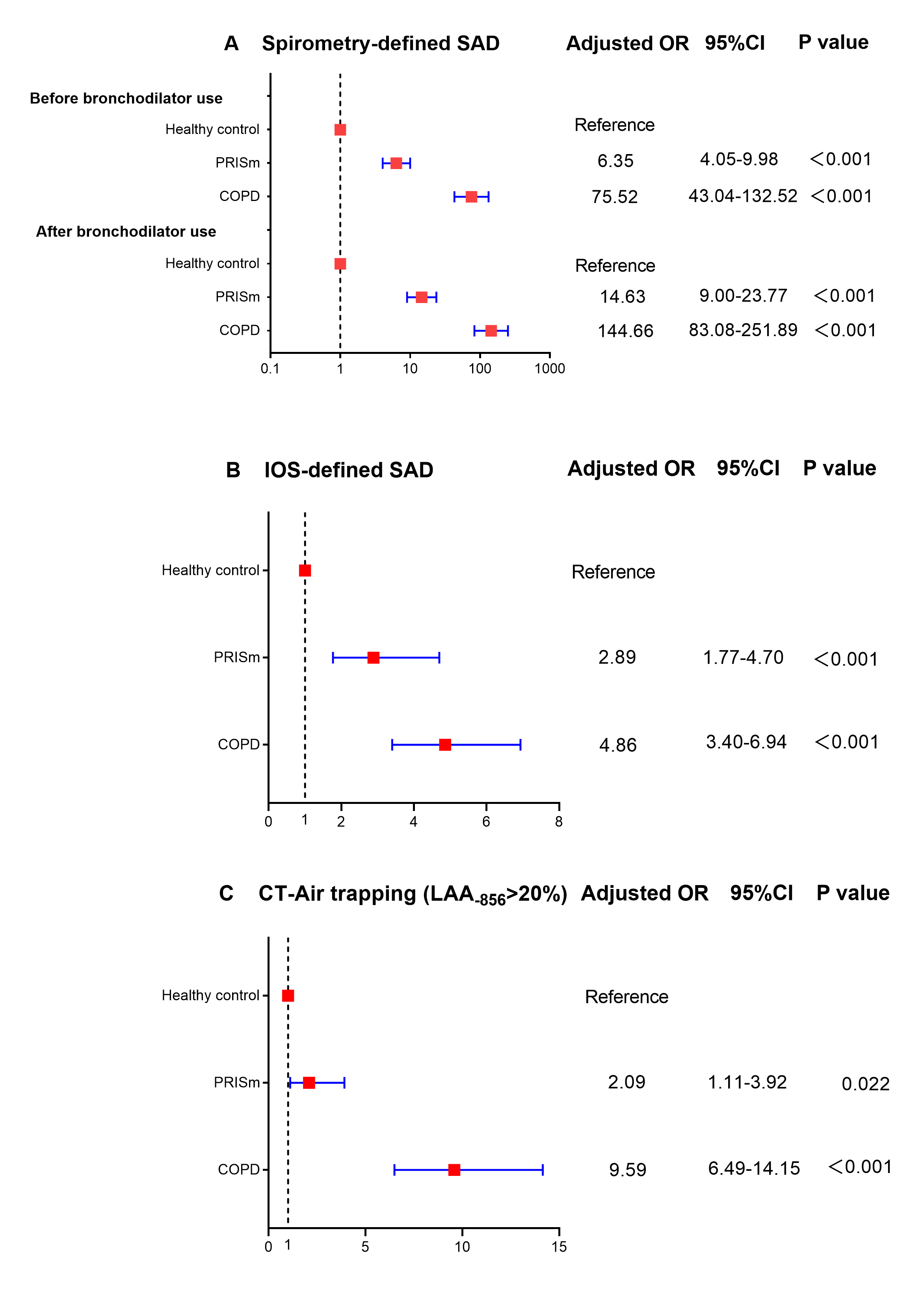


Abbreviations: PRISm, preserved ratio impaired spirometry; SAD, small airway dysfunction; OR, odds ratio; CI, confidence interval; GOLD, Global Initiative for Chronic Obstructive Lung Disease; LAA_-856_, low-attenuation area of the lung with attenuation values below -856 Hounsfield units.

Analyses were adjusted for age, sex, body mass index, smoking status, and smoking index.

P value is a result of comparison with the healthy control group.

**Figure E6**. Effect of PRISm on small airway dysfunction parameters expressed as odds ratio and 95% confidence intervals in subgroups with TLC_CT_≥70% of the predicted value.


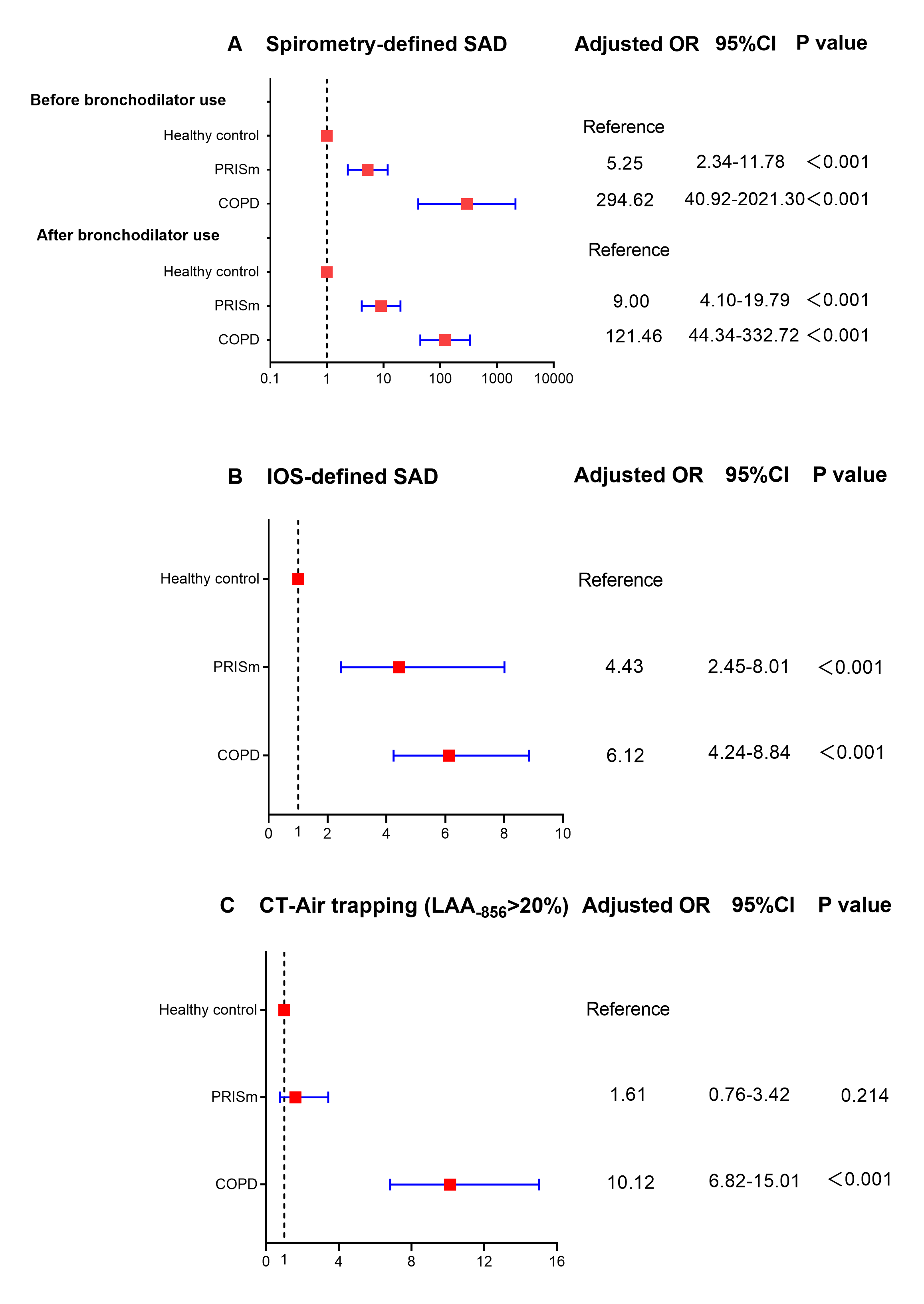


Abbreviations: PRISm, preserved ratio impaired spirometry; SAD, small airway dysfunction; OR, odds ratio; CI, confidence interval; GOLD, Global Initiative for Chronic Obstructive Lung Disease; LAA_-856_, low-attenuation area of the lung with attenuation values below -856 Hounsfield units.

Analyses were adjusted for age, sex, body mass index, smoking status, and smoking index.

P value is a result of comparison with the healthy control group.
